# Nanopore Trap for Label‐Free Fingerprinting of Surface‐modified Single Nanoparticles

**DOI:** 10.1002/smtd.202501765

**Published:** 2025-12-09

**Authors:** Nianduo Cai, Tzu‐Heng Chen, Yunfei Teng, Akhil Sai Naidu, Aleksandra Radenovic

**Affiliations:** ^1^ Laboratory of Nanoscale Biology, Institute of Bioengineering École Polytechnique Fédérale de Lausanne (EPFL) Lausanne CH‐1015 Switzerland; ^2^ NCCR Bio‐Inspired Materials École Polytechnique Fédérale de Lausanne (EPFL) Lausanne CH‐1015 Switzerland

**Keywords:** nanopore trapping, single‐particle sensing, solid‐state nanopore

## Abstract

Label‐free characterization of nanoparticle surface functionalization at single‐particle resolution is essential for a wide range of applications. Solid‐state nanopore sensing provides a direct electrical readout that is intrinsically sensitive to the size, surface layer, and interfacial chemistry of single particles in liquid environments. The trapping‐based nanopore sensing regime further enables probing surface‐dependent particle‐pore interactions with extended observation time. Here, a solid‐state nanopore trap‐based fingerprinting method is presented to differentiate single nanoparticles with distinct surface modifications. The method combines a “trap‐release” measurement protocol with a multi‐metric analysis workflow that extracts blockade distributions, sub‐level statistics and frequency‐domain signatures from trapping events, and constructs a unique fingerprint for each particle species. Applied to silica cores (≈25–30 nm) functionalized with APTES, NHS‐PEG_4_‐Biotin and Tween‐20, the approach generates distinct fingerprints that map to surface charge, coating conformation and configuration heterogeneity. Moreover, in situ detection of surface chemical transformation via specific streptavidin binding is demonstrated, with stoichiometry‐dependent progression of the fingerprints. This platform provides a complementary tool to optical, spectral and ensemble assays for characterizing engineered nanoparticle surfaces and tracking interfacial molecular interactions in solution with label‐free and single‐particle sensitivity.

## Graphical abstract

A solid‐state nanopore trapping‐based methodology combines “trap‐release” measurement protocol with multiple features extracted from ionic current of trapping events to generate unique electrical fingerprints of nanoparticles with distinct surface functional layers. Applying this methodology enables the differentiation between APTES, PEG_4_‐Biotin and Tween‐20‐coated silica nanoparticles, and to monitor in situ surface chemical transformation via specific streptavidin binding on biotinylated nanoparticles.

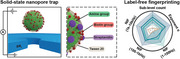

## 1 Introduction

Surface functionalization shapes nanoparticle properties such as stability, specificity, and interaction potential, making its accurate characterization a critical step across diverse scientific and technological applications.^[^ ^1^, ^2^, ^3^ ^]^ Functionalizations (like covalent bonding,^[^ ^4^, ^5^ ^]^ biomolecule conjugation,^[^ ^6^, ^7^ ^]^ or surfactants physisorption,^[^ ^8^, ^9^ ^]^ etc.) have provided distinct chemical structures and physical properties to the nanoparticle surfaces, such as surface charge, thickness and mechanical rigidity of the coating layer, local roughness and conformational entropy, etc. These surface layers dominate the colloidal stability, electrostatic interactions, steric flexibility and interfacial behaviors of nanoparticles, thereby controlling how they interact with the environment.^[^ ^2^, ^3^ ^]^ Given the significance of knowing how these microscopic surface characteristics determine nanoparticle behaviors, an accurate, label‐free characterization method at single‐particle, in‐liquid level becomes essential for validating the nanoparticle design and ensuring reproducible performances.

A broad spectrum of state‐of‐the‐art single‐particle analytical techniques have been employed in recent years, to probe different physical observables and understand engineered nanoparticle behavior. For example, cryogenic electron microscopy (Cryo‐EM) has been shown to provide near‐atomic to nanometer resolution for nanoparticle morphology and conformational heterogeneity.^[^ ^10^, ^11^ ^]^ Optical microscopy‐based techniques can deliver information about in‐liquid dynamics,^[^ ^12^ ^]^ and other label‐free optical methods like interferometric scattering microscopy (iSCAT) measure optical/mass contrast and is used to analyze size and trajectories or various nanoparticles;^[^ ^13^ ^]^ enhanced Raman microscopies provide direct identifications of surface ligands with single‐particle sensitivity via the engineered hotspots;^[^ ^14^, ^15^ ^]^ and single‐particle inductively coupled plasma mass spectroscopy (spICPMS) detects elemental chemical composition of noncarbon nanomaterials as well as their number concentration, size and the number size distribution.^[^ ^16^, ^17^ ^]^ However, when used to differentiate subtle differences on nanoparticle surface functional layers, these methods either require specific labeling or are limited in sample conditions, such as vacuum or fixation. Therefore, there still remains the need for a single‐particle characterization method that is label‐free, operable in liquid, and capable of tracking surface chemistry and surface heterogeneity‐related interactions under well‐controlled experimental conditions with an unambiguous physical readout.

Solid‐state nanopore (SSN) sensing offers a complementary electrical approach to bridge this analytical gap. Such nanopore is a nanometric aperture in a solid‐state membrane (such as silicon nitride, SiN_x_) that separates the two compartments filled with ionic solutions. With the pore serving as the only channel of ionic current flow, any analyte (such as biomolecule, or nanoparticle) interacting with the nanopore, either translocating or residing within the sensing region, causes a modulated current signal, which is characteristic of the analyte. The ionic current signal contains rich, multi‐dimensional features such as the blockage amplitude, dwell time, and frequency‐domain signatures in current fluctuations. These features reflect information about the size, shape, mobility and surface properties of the target, making nanopore electrical readouts a powerful complementary metrology.^[^ ^18^ ^]^ Therefore, SSN has been widely used for label‐free single‐molecule (such as DNA,^[^ ^19^, ^20^ ^]^ proteins^[^ ^21^, ^22^ ^]^ and glycans,^[^ ^23^, ^24^ ^]^ etc.) and single‐nanoparticle analysis,^[^ ^25^, ^26^, ^27^ ^]^ addressing the ensemble‐average limitation and providing multi‐dimensional, analyte‐specific information. While analytes interact with the pore, trapping‐based sensing, which utilizes nanopore with a diameter smaller than that of the analytes, offers unique advantages over transient translocation. By trapping analytes near the nanopore orifice, this method extends the observation time of the analyte and provides temporal information across wider timescales, enabling it to reveal subtle differences in analyte conformational states.^[^ ^22^, ^28^, ^29^, ^30^ ^]^ Specifically, the nanopore electro‐osmotic trap (NEOtrap) developed by Schmid et al. creates a DNA‐origami‐docked nanocavity with lipid layer‐coated SSN, which confines single proteins for multiple hours, enabling a label‐free observation of protein conformational heterogeneity.^[^ ^22^ ^]^ Previous works have differentiated nanoparticles via electrical trapping on nanopores, which are mainly based on the size‐ or surface charge‐dependent current blockage levels.^[^ ^31^, ^32^ ^]^ However, beyond steady‐state blockage amplitudes, extended electrical features that are provided by the trapping‐enabled rich signals could further enhance the sensitivity to surface chemistry, facilitating the differentiation of nanoparticles when the cores are similar but surface functional layers differ subtly.

**Figure 1:**
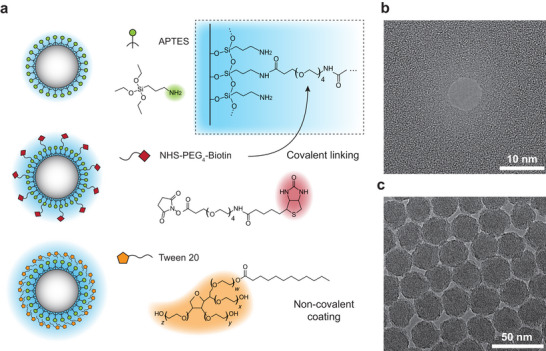
a) Schematic representation of the nanoparticles with three different surface functionalizations, using APTES, NHS‐PEG_4_‐Biotin and Tween‐20. Amine groups (─NH_2_, in green) are first covalently primed on the silica nanoparticle core (in white), resulting in SiO_2_─NH_2_ nanoparticle. Subsequent biotinylation on the amine groups leads to partially modified surfaces with PEG arms and biotin (in red) terminates. Insets depict the covalent silane linkage (top right). Non‐covalent coating of Tween‐20 forms a physisorbed micellar shell around the SiO_2_─NH_2_ nanoparticles. The hydrophilic polyethylene oxide chain and sorbitan headgroup are noted (in yellow). Transmission electron microscopy (TEM) image of b) a typical silicon nitride (SiN_x_) nanopore used in the study, and c) self‐synthesized silica nanoparticles. The solid‐state nanopores used in the study have a general range of sizes between 6–10 nm in diameter, and the silica nanoparticles have a size distribution between 25–30 nm in diameter.

Therefore, in this work, we present a SSN trap‐based label‐free fingerprinting methodology to systematically profile nanoparticle surface functional layers. Single nanoparticles were trapped on SiN_x_ nanopore by leveraging the electrophoretic (EP) force, electro‐osmosis flow (EOF), and supporting force from the nanopore membrane. The surface‐dependent pore‐particle interaction currents were analyzed as differentiable fingerprints. We first demonstrated the reversible nanopore trap with an electrical trap‐release measurement protocol. Such design offers three advantages: 1) a trapping‐enabled long observation time that captures processes across broad timescales, 2) sufficient blockade signals that detect subtle surface modifications, and 3) repeated single‐particle measurements for statistical analysis enabled by capture‐dock‐release cycles. From the ionic current signals of each nanoparticle trapping event, an analysis pipeline was developed to extract multi‐dimensional metrics, including blockade amplitudes, sub‐level statistics, and frequency‐domain signatures from current fluctuations. These metrics together constitute the particle's electrical fingerprint. Then, we applied the methodology to three systematically modified silica nanoparticles with similar core size (27 ± 2 nm), i.e., (3‐Aminopropyl)triethoxysilane (APTES)‐aminated (─NH_2_), biotinylated by NHS‐PEG_4_‐Biotin, and Tween‐20 surfactant coated (**Figure** **1**a), and obtained distinct electrical fingerprints for each type of surface functional groups. We further demonstrated in situ monitoring of surface chemical transformation, specifically via the binding of streptavidin to biotin‐functionalized nanoparticles under different stoichiometric ratios, and each binding stage produces a unique and progressively shifting fingerprint. Combined with the trap‐based sensing and multi‐metric analysis, the SSN platform thus provides a practical, label‐free complementary methodology that reports single‐particle surface heterogeneity and in‐liquid dynamic signatures, ensuring its efficacy in future applications.

## 2 Results and Discussion

### 2.1 Single‐Nanoparticle Trap on Solid‐State Nanopore

We first demonstrate the trapping of nanoparticles at the nanopore orifice using a solid‐state nanopore (SSN) (Figure 1b) with a diameter smaller than that of the particles (Figure 1c). The nanopore conductance is initially measured in 1 M KCl solution and compared with the TEM characterization to confirm the pore's working condition. Once nanoparticles are introduced into the cis chamber of the flow cell and captured by the nanopore, a change in ionic conductance will be observed, indicating local geometrical changes due to interactions between the particle and the pore. Full translocation of nanoparticle is prohibited due to its larger diameter compared to the pore, therefore setting the system in a trapping regime, where particles are confined in the vicinity of the nanopore. This configuration defines the operational mode of trapping‐based nanopore sensing.

With this sensing configuration, the nanoparticle is constrained to the position on nanopore, eliminating the variability associated with fast translocation. The long and stable current blockade signals enable low‐pass filtering to reduce noise and therefore improve the SNR.^[^ ^33^, ^34^ ^]^ In the meantime, it provides an extended temporal window to probe the interactions between nanoparticle and nanopore surfaces.^[^ ^22^, ^29^ ^]^ Following this concept, we designed the single‐nanoparticle trap on SiN_x_ nanopore by using nanopores with diameters that are substantially smaller than nanoparticles. In the meantime, to enhance the sensitivity of ionic blockage to subtle changes on nanoparticle surface, the contribution to ionic conductance perturbation from the surface functional layer should be significant compared with that from the particle bulk, meaning that the particle size should not be too large to maintain a considerable surface‐to‐volume ratio. Given these considerations to enable constant trapping and improve the sensitivity to surface functionalization layers, we used SiN_x_ nanopore mostly between ≈6–10 nm in diameter in this study (Figure S1 and Table S1, Supporting Information), to trap nanoparticles that are between ≈25–30 nm in diameter (Figure S2, Supporting Information).

**Figure 2:**
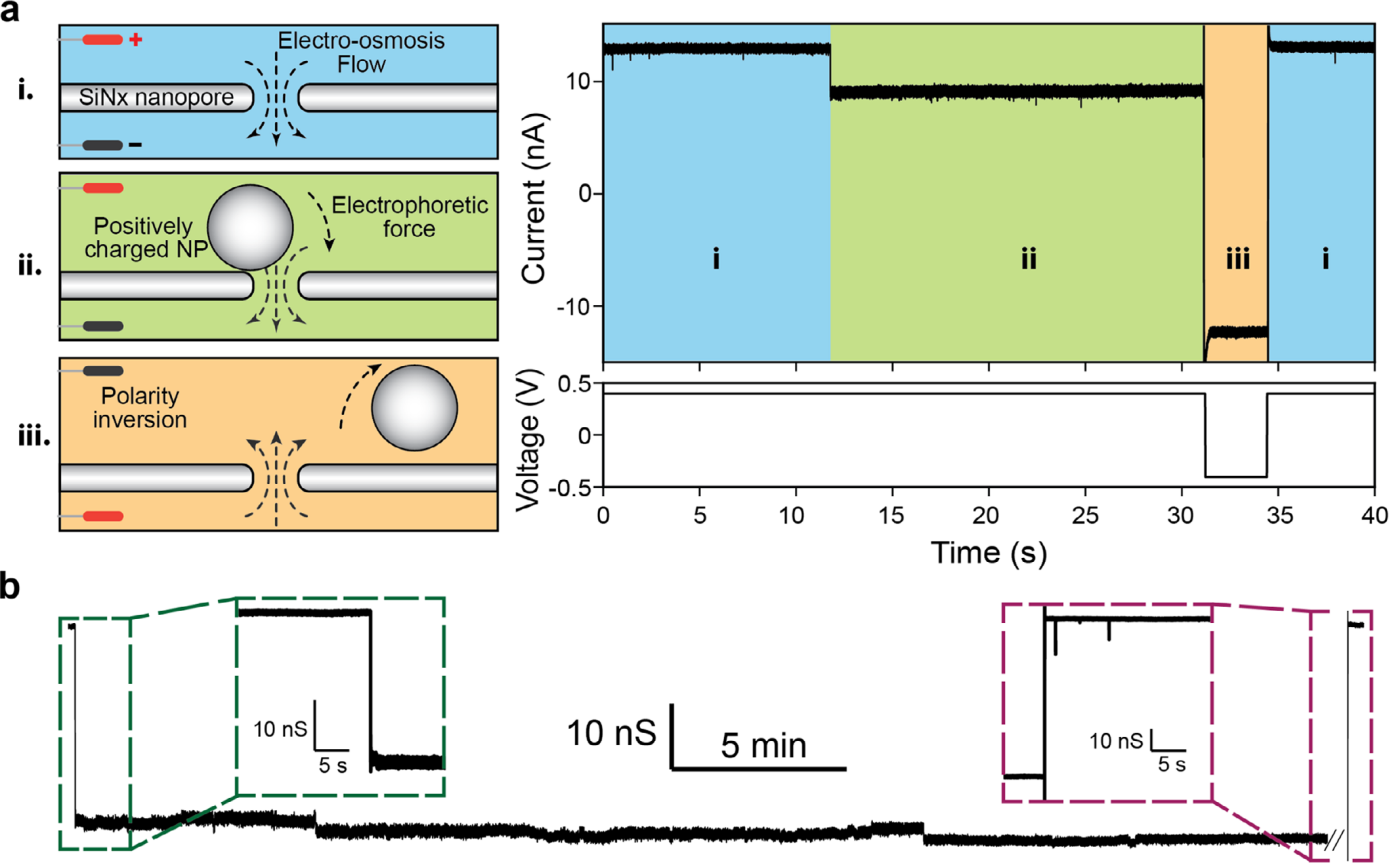
a) Schematic illustration of different regimes in one single nanoparticle gating event and corresponding ionic current recording as well as voltage profile. Each gating event starts from regime i) an “open” nanopore state with a stable baseline current (red), which also induces an electro‐osmosis flow (EOF) to facilitate nanoparticle capturing; ii) a positively charged nanoparticle docks on the nanopore due to simultaneous EOF and electrophoretic (EP) force, while supported by the membrane, leading to a “gated” nanopore state with corresponding current blockage (blue); iii) the nanoparticle is released from the nanopore by reversing the polarity of applied voltage (purple), returning to the “open” nanopore state (red). b) A long gating event of SiO_2_‐Tween nanoparticles was recorded for over 30 min, and the nanopore returned to “open” state by manually reversing the voltage. A constant voltage at 300 mV was applied since the start of measurement, and open‐pore baseline current at 13.7 nA indicated the nanopore diameter d_pore_=8.7 nm. The current drops to ≈3 nA after trapping the nanoparticle. To release the particle and return to open‐pore state, the voltage was reversed to −300 mV. Insets show zoom‐in views of the start of nanoparticle gating (green) and end of the event (dark red) after reversing the voltage. The unprocessed data of full recordings are shown in Figure S3 (Supporting Information).

The capturing, trapping and releasing of a nanoparticle at the pore orifice are enabled by the EOF and EP forces, as illustrated in **Figure** **2**a. Under an applied voltage, EOF is generated due to the negatively charged pore surface, which produces a hydrodynamic drag force that can assist the capture of nanoparticles in the solution (Figure 2a, regime i).^[^ ^35^, ^36^, ^37^ ^]^ The “open‐pore” state is characterized by the stable current baseline corresponding to the expected ionic conductance. For the case of a positively charged nanoparticle, besides EOF, the EP force that acts on the particle's surface charge also facilitates the capture. Once the nanoparticle is docked on the nanopore surface under both forces, a supporting force from the membrane balances the EOF and EP force and stabilizes the trapping of particle. The trapping leads to an ionic conductance blockade, which in turn characterizes electrically the particle‐pore interactions (“gated‐pore” state, regime ii). We further validated the trapping efficacy with a constant and stable single‐particle trapping event for over 30 min (Figure 2b). Similar to other established nanopore trap,^[^ ^22^ ^]^ our SSN trap supports long and stable gating events that provide sufficient temporal window for the fingerprinting of nanoparticles with various surface functionalization layers. In addition, the EOF and EP force‐assisted trap also enables reversible sensing capability,^[^ ^38^, ^39^ ^]^ which is realized by inverting the external voltage and therefore reversed EOF and EP force (Figure 2a, regime iii). It restores the “open‐pore” state (regime i) of the sensing system, making the SSN ready for the next trapping event. This feature allows acquisition of repeated blockade events on a single‐particle level and facilitates statistical analysis of the interactions between the particle and nanopore. While showing efficient trapping capability, our method presents a simpler setup of the trap compared to another reported trap,^[^ ^22^ ^]^ where EOF and EP‐force are leveraged to generate a nanocavity, enabling the repeated independent trap‐release cycles. Accordingly, the different trapping setup leads to different selectivity on analyte size, as our method is capable of trapping nanoparticles larger than the nanopore size, while the nanocavity prefers single analytes which have slightly smaller size than that of the nanopore.

### 2.2 Trap‐Release Voltage Cycling and Multi‐Metric Analysis

**Figure 3:**
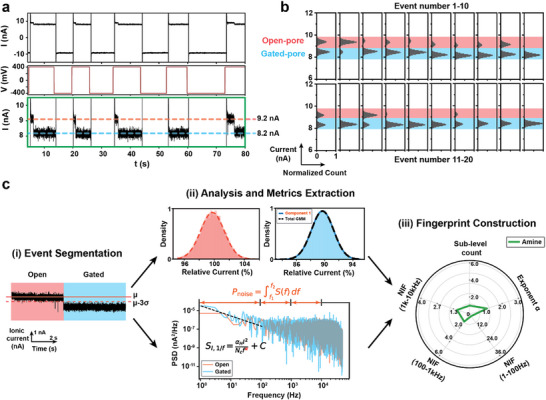
Illustration of nanoparticle trapping‐based nanopore methodology, using SiO_2_─NH_2_ nanoparticles for demonstration. a) Voltage stimuli and ionic current trace for 5 continuous cycles of trapping events. The inset (green box) shows a zoom‐in view of the current trace in these trapping events with clear indication of open‐pore (red) and gated‐pore (blue) states. b) Histogram of 20 continuous gating events. c) Demonstration of the analysis workflow, from step i) extraction and segmentation of each event into “open” and “gated” regions, then step ii) extraction of features by various quantitative analysis in both regions, and step iii) construction of the nanoparticle fingerprint with multiple features. The event segmentation is realized based on a threshold‐detection algorithm. Gaussian mixture model (GMM) is applied to fit the histogram of relative current in each segment, and the number of sub‐level components are extracted based on optimal fitting results. The exponent ɑ from low‐frequency regime is extracted via fitting the power spectral density (PSD) in 1–100 Hz regime. Noise increase factors (NIFs) are obtained by first computing the specific band‐integrated PSD and then calculating their ratios between the “gated” and “open” segments. Detailed analysis workflow is described in the Experimental Section.

Exploiting the voltage‐reversible nature of the trap, in which the inversion of the applied bias reverses the EOF and EP forces, thereby releasing the trapping nanoparticle, we establish a “trap‐release voltage cycling” measurement protocol and an analysis workflow with multiple metrics (**Figure** **3**). In each voltage cycle, a positive voltage is first applied and the nanoparticle is trapped on the nanopore, which is characterized by a transition in ionic currents. The external voltage is then reversed to release the nanoparticles from nanopore orifice, indicated by the restored current to open‐pore state. By repeating such a trap‐release voltage cycle, we obtain sufficient electrical signals from each nanoparticle's gating events. As each cycle results in electrical signals containing open‐ and gated‐pore segments, they are first separated by applying a 3σ_*open*_ threshold, where σ_*open*_ represents the root‐mean‐square (RMS) noise from the open‐pore baseline current. After segmentation, we first use open‐pore segment current to evaluate the nanopore condition and screen events with baseline drift. For events collected under normal working conditions, the gated‐pore segments are further analyzed to extract features from the current signal. We extract first the sub‐level components via fitting with Gaussian Mixture Model (GMM), and then derived frequency‐domain signatures from the power spectrum density (PSD), including the low‐frequency exponent α from 1/f fit over 1–100 Hz, and noise increase factors (NIFs), defined as the ratio of band‐integrated PSD in 1–100 Hz, 100–1k Hz, and 1–10 kHz bands between gated‐ and open‐pore signals. The combined features form a multi‐metric radar‐like electrical fingerprint that is characteristic of each nanoparticle's surface functional layer.

A detailed demonstration of this measurement protocol is shown in Figure 3a with five consecutive trap‐release cycles of SiO_2_─NH_2_ nanoparticles. This protocol provides many independent and in‐solution snapshots of the particle‐pore interactions. The ionic current distributions of 20 consecutive trapping events from SiO_2_─NH_2_ nanoparticles are summarized with a histogram (Figure 3b). The narrow and consistent distribution of open‐pore current (red) confirms the experimental stability, indicating no baseline drift or pore contamination, which underscores that each gating event is a reliable representation of the nanoparticle surface chemistry and the particle‐pore interactions. This enables a robust dataset generation for subsequent feature extraction. Additionally, the blockade amplitude (blue) is summarized through a histogram, which directly shows its distribution among multiple individual events. Here, the gated currents converge to a stable distribution around a single level, which indicates the highly reproducible trapping behaviors of the SiO_2_─NH_2_ nanoparticles on the nanopore. Moreover, the blockade histograms bridge the raw data and the following multi‐metric analysis. As shown in Figure 3c, we first extract each trapping event according to the voltage application timepoint of the trap‐release cycle. A threshold of three‐fold RMS of open‐pore baseline current, which is recorded after checking the nanopore condition and before adding nanoparticle samples, is usually applied to separate the gated‐pore segment out from the event (Step i). This threshold helps avoid false‐positive gating events, which may occur due to baseline fluctuations and fall within the three‐fold threshold. After extracting the gated‐pore segments, we performed GMM fitting to identify the sub‐level components (step ii, top): for open‐pore segment, ideal number of components should be 1 to validate the stability of baseline current; while for the gated‐segment, number of sub‐level components is extracted to quantify the complexity of particle‐pore interaction, as a higher sub‐level value possibly indicates a more dynamic and heterogeneous interaction. For the SiO_2_─NH_2_ nanoparticle, the optimal GMM fitting results in only one sub‐level component, which is consistent with the single distribution of gating current levels in the histogram. This clearly represents the non‐complex interactions between the nanoparticle and nanopore and could therefore reflect the characteristic of the surface functional layers on nanoparticles. This work examines examples of nanoparticles with more complex surface chemical structures, where the histograms display a broader and multi‐modal distribution, and the GMM fitting resolves multiple sub‐level components (details in Figure S4, Supporting Information).

Besides the sub‐level components, we further extracted other features from the frequency‐domain signatures of each gating event (step ii, bottom), as these signatures from current fluctuations also infer physical processes happening at the pore.^[^ ^40^, ^41^ ^]^ For this purpose, power spectral density (PSD) is usually adapted to analyze ionic current fluctuations and to interpret changes in each frequency domain.^[^ ^42^ ^]^ We computed the PSD for both open‐ and gated‐pore segments using Welch's method.^[^ ^43^ ^]^ Based on the different sources of current fluctuation,^[^ ^42^, ^44^ ^]^ we decompose the spectrum and extract the low‐frequency (1–100 Hz) exponent ɑ and the noise increase factors (NIFs) over discrete frequency bands (1–100 Hz, 100–1k Hz, and 1–10 kHz). The low‐frequency noise, or often referred to as “flicker” noise or 1/f noise, typically dominates in the 1–100 Hz range. It is known to be associated with the slow physical process such as the fluctuation of charge carriers near the nanopore. The exponent ɑ is specifically linked with the fluctuating processes at the surface and their temporal dynamics,^[^ ^45^, ^46^ ^]^ therefore can be used as a sensitive characteristic for the interactions between different nanoparticle surfaces and nanopores. To extract the exponent ɑ, we fit the low‐frequency component in PSD, following the description of 1/f power^[^ ^47^ ^]^ as

(1) $$S_{I,1/f}=\frac{\alpha_{H}I^{2}}{N_{C}f^{\alpha }}+C$$

where α_*H*_, the Hooge's constant, is an empirical parameter that quantifies the magnitude of the 1/f noise fluctuations, *I* is the ionic current, *N_C_* is the number of charge carriers in the nanopore volume and *C* is a constant that takes consideration of the frequency‐independent white noise contribution, as discussed in previous work.^[^ ^46^ ^]^ Meanwhile, since the nanoparticle surface functionalization affects the local ionic flow and dielectric environment when the particles interact with the nanopore orifice, the measurable changes in PSD are not limited to low‐frequency 1/f component, but also exist across distinct frequency domains.^[^ ^40^, ^44^, ^48^ ^]^ To capture these effects and use them as characteristic signatures of each particle‐pore interaction, we compute band‐integrated PSD over 1–100 Hz, 100–1k Hz, and 1–10 kHz and express their ratios between gated‐ and open‐pore segments as noise increase factors, or NIFs, which are used as the additional features in our multi‐metric workflow. The calculation of NIF is described as:

(2) $$NIF=\frac{P_{noise,gated}}{P_{noise,open}}=\frac{\int_{f_{1}}^{f_{2}}S_{I,gated}\left(f\right)df}{\int_{f_{1}}^{f_{2}}S_{I,open}\left(f\right)df}$$

where *P_noise_* represents the band‐integrated PSD value and *S_I_* represents noise power in different frequency domains.

By combining the GMM‐derived sub‐level components with the frequency‐domain signatures, we thereby create a compact multi‐metric fingerprint (Figure 3c, step iii) that quantitatively discriminates nanoparticles with different surface functionalizations.

### 2.3 Differentiation of Surface‐Functionalized Nanoparticles via Nanopore Methodology

**Figure 4:**
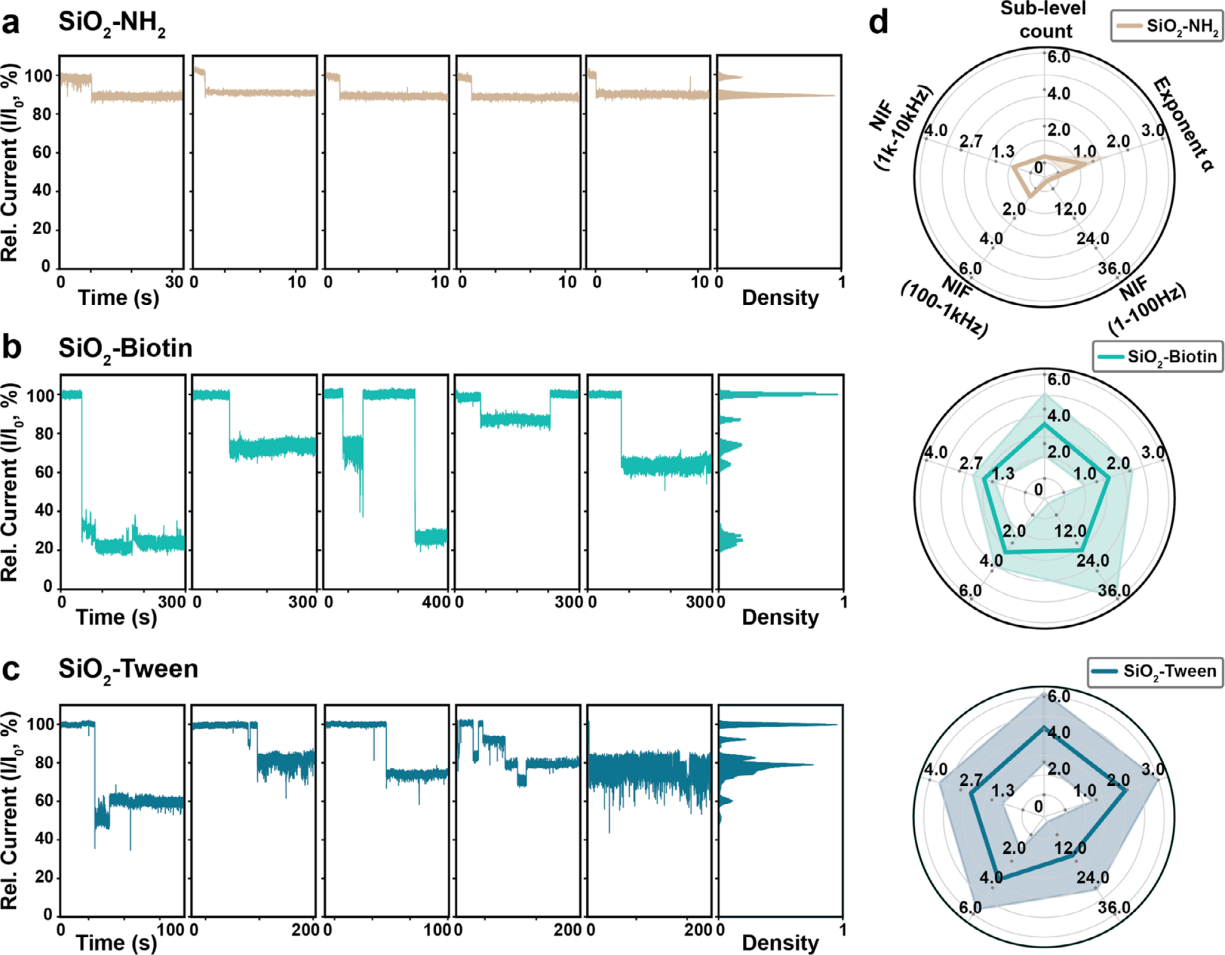
Differentiation of three types of nanoparticles with different surface functionalizations using the nanopore‐enabled methodology. a–c) Representative normalized ionic current traces (left) with corresponding histograms (right) showing blockade‐level distributions for three types of nanoparticles. a) The SiO_2_─NH_2_ nanoparticles exhibit shallow (≈10%) and stable single‐level blockages. b) SiO_2_‐Biotin nanoparticles show generally deeper (≈20%–80%) blockages with discrete multiple sub‐levels. c) SiO_2_‐Tween nanoparticles display more dynamic and flickering trapping behaviors with a broad and noisy blockage distribution (≈20%–60%), without well‐defined discrete levels. All events were originally collected with 200 kHz sampling rate and low‐pass filtered to 50 kHz. The current is normalized by the “open” pore current I_0_ in each event for better visualization of the blockage levels. Histograms combine all relative current I/I_0_ from the characteristic events. d) Radar‐plot fingerprints for each type of nanoparticle. The solid line in each plot represents the mean value of each feature, with the colored band representing one standard deviation around the mean. For each type of nanoparticles, the measurements are repeated on different nanopore devices (*n* ≥ 2) (Table S1, Supporting Information). Experiment results from device BC_48 (d_pore_ = 5.7 ± 0.1 nm), BC_52 (d_pore_ = 7.4 ± 0.2 nm) and BC_62 (d_pore_ = 6.8 ± 0.1 nm) are analyzed to represent SiO_2_─NH_2_, SiO_2_‐Biotin and SiO_2_‐Tween nanoparticles, respectively. To compute the radar plot metrics, the numbers of nanoparticle trapping events used for each type of nanoparticles are *n*(SiO_2_─NH_2_) = 21, *n*(SiO_2_‐Biotin) = 15, and *n*(SiO_2_‐Tween) = 21. The detailed values of radar plot metrics are provided in Table S2 (Supporting Information).

To validate this methodology, we collected the trapping events from silica nanoparticles that are modified with three distinct surface functional layers, including APTES (SiO_2_─NH_2_), NHS‐PEG_4_‐Biotin (SiO_2_‐Biotin) and Tween‐20 (SiO_2_‐Tween), and applied the fingerprinting workflow to examine how these surface layers modulate nanoparticles gating behavior at the nanopore (**Figure** **4**). We first measured each type of nanoparticle on individual devices (details in Table S1, Supporting Information) in the 1 M KCl solution, using the trap‐release cycling protocol. The collected raw data of ionic currents were processed according to the workflow to extract valid gating events and construct multi‐metric fingerprints for each type of nanoparticles (more details in the Experimental Section).

Figure 4a–c show the representative trapping events and corresponding blockage histograms for each type of nanoparticles. The SiO_2_─NH_2_ particles produce predominantly stable gating current traces with shallow blockage levels (≈10%) and single, narrow peak in the histogram (Figure 4a). By contrast, the SiO_2_‐Biotin particles exhibit generally deeper blockage levels (≈20%–70%), with several discrete conductance levels appearing in the histogram (Figure 4b). SiO_2_‐Tween particles exhibit similarly broad and highly fluctuating blockage levels (≈10%–60%), with a smeared distribution in the histogram and no well‐defined peaks (Figure 4c). GMM analysis across all gated‐pore segments shows the same trend and quantifies these differences, as the average sub‐level count per event increased from ≈1 for SiO_2_─NH_2_, to ≈3 for SiO_2_‐Biotin and ≈4 for SiO_2_‐Tween nanoparticles (Figure 4d) (details in Table S2, Supporting Information).

The blockage distribution and sub‐level results together point to distinct patterns of nanoparticle‐nanopore interactions, which are linked to the different surface functional layers of each nanoparticle species. The narrow and single‐level blockades of SiO_2_─NH_2_ represent a stable and monotonic trapping behavior, consistent with what might be expected from a compact and covalently bound silane film.^[^ ^49^, ^50^, ^51^ ^]^ Such mechanically rigid and uniform coating would exhibit limited configuration variabilities at the pore orifice, and produce a temporally stable perturbation to the ion flow. The stable trapping of SiO_2_─NH_2_ nanoparticles was also observed in DNA translocation experiments (Figure S5, Supporting Information). Alternatively, the discrete and multiple‐level current blockages of SiO_2_‐Biotin particles suggest that the trapping takes several specific configurations. This is possibly due to the fact that the PEG_4_‐Biotin coating can adopt a small set of metastable conformations,^[^ ^52^, ^53^ ^]^ and therefore several geometrical configurations occur when particles dock on the pore. Each distinct configuration between the nanoparticle surface layer and the nanopore corresponds to a different pore conductance. The smeared and highly variable blockades of SiO_2_‐Tween particles reflect a more unstable and heterogeneous interaction between the particle surface and nanopore. This could likely be attributed to the altered interfacial properties (such as surface energy) resulting from the surfactant effect of Tween‐20,^[^ ^54^, ^55^ ^]^ which destabilizes nanoparticle trapping, or the labile and reconfigurable structure of Tween‐20 under perturbations,^[^ ^8^, ^56^ ^]^ such as electrical field stimuli in nanopore confinement. A continuum of transient configurations may appear during trapping, rather than well‐defined docking states.

Frequency‐domain analysis of gated‐ and open‐pore segments revealed consistent and surface layer‐dependent contrasts across the nanoparticle species. The fitted low‐frequency exponent α increased progressively from SiO_2_─NH_2_, to SiO_2_‐Biotin and SiO_2_‐Tween nanoparticles. In the meantime, band‐integrated PSD ratios, or NIFs also showed notable increases for SiO_2_‐Biotin and SiO_2_‐Tween in all frequency bands when comparing with the SiO_2_─NH_2_ nanoparticles.

These trends provided information complementary to the sub‐level analysis. As the low‐frequency exponent reflects temporal information and complexity of interfacial processes at the pore, the larger α values suggest slower and more complex surface processes between the nanoparticle surface functional groups and the nanopore.^[^ ^45^, ^46^ ^]^ This aligns with our discussion about the structural features of different surface layers. The short, covalently‐bound silane film would present fewer conformational fluctuations and less long‐timescale perturbations to the ionic flow compared with the longer and more flexible ethylene glycol chain in PEG_4_ (e.g. (─CH_2_─CH_2_─O─)_4_) and multiple ethylene glycol chains in Tween‐20. The pronounced increase of band‐integrated PSD in the low frequency range (1–100 Hz) for PEG_4_‐Biotin and Tween‐20‐coated particles also suggests the presence of slow interfacial processes during trapping. We also measured the trapping of Tween‐20 coated SiO_2_‐Biotin nanoparticles (Figure S6, Supporting Information). The current fluctuation, blockage levels and frequency‐domain signatures exhibit similar trends to those of the SiO_2_‐Tween particles, indicating that the physisorbed Tween‐20 coating introduces similar features to both nanoparticles. The mid‐frequency band (100 Hz–1k Hz) PSD is normally dominated by the conductance‐dependent white noise.^[^ ^44^ ^]^ Trapping‐induced conductance reduction would be expected to lower the mid‐band power and yield NIF smaller than 1. Instead, we observed NIFs all larger than 1, and they increase gradually from SiO_2_─NH_2_ to SiO_2_‐Tween particles. Possible contribution of the mid‐band power may come from shot noise, which originally arises from potential barriers in ionic flow paths.^[^ ^42^ ^]^ The surface layers could introduce extra ionic obstacles and barrier‐related fluctuations when nanoparticles are trapped at the pore orifice, thereby causing higher noise in this range. Similar results were shown in previous work,^[^ ^39^, ^57^ ^]^ while further studies are required for better understanding. Changes in high‐frequency band (1–10 kHz) PSD are associated with the dielectric loss or localized leakage current.^[^ ^42^, ^44^ ^]^ The increases in this regime could therefore be attributed to the polarizable surface groups, which reorient under the applied field, thus reducing the dielectric insulation and bringing in pathways for charge carriers. While a clear insight of the mechanism requires follow‐up, such as studies on the dielectric properties of surface functionalized nanoparticles, etc.

As the nanoparticle trapping experiments are studied on nanopores with different sizes, here we also discuss how the pore size influences the trapping behaviors and the fingerprinting metrics. Because the nanoparticles (≈27 nm core size) are much larger than the pores used here, trapping is mainly controlled by the leakage path between gated particle and nanopore orifice, making the current signals more sensitive to the nanoparticle surface layer and its interaction with the pore. Therefore, larger pores lead to larger leakage gaps, resulting in less conductance modulation and permitting more complex configurational changes of the nanoparticle when trapped at the pore orifice, as observed in the results when comparing the trapping of same Tween‐20‐coated SiO_2_‐Biotin nanoparticles on two distinct nanopores (≈9 nm vs. ≈13 nm) (Figure S7, Supporting Information). Besides pore size, the membrane thickness also affects the trapping behaviors as it changes the electric field distribution. Similarly, the electric field affects the configurational changes of nanoparticles in the gated‐state and thus the metrics. We studied the effects of electric field via changing the applied voltages on two types of nanoparticles (Figures S8–S10, Supporting Information), the results reveal that the effect is also surface layer‐dependent.

Overall, the frequency‐domain features provide complementary insights into the physical interpretation of nanoparticle‐pore interactions, in addition to the direct current blockage and sub‐level analyses. We also studied the trapping behavior of SiO_2_‐Biotin and Tween‐20‐coated SiO_2_‐Biotin nanoparticles with the methodology under different voltages, and the results further support that the fingerprinting method reveals subtle differences between nanoparticles based on surface layer (Figures S8–S10, Supporting Information). Together, the surface chemistry‐dependent trapping behaviors enable the use of these multi‐metric fingerprints to differentiate between APTES, PEG_4_‐Biotin and Tween‐20 modified silica nanoparticles.

### 2.4 In Situ Detection of Streptavidin Binding

**Figure 5:**
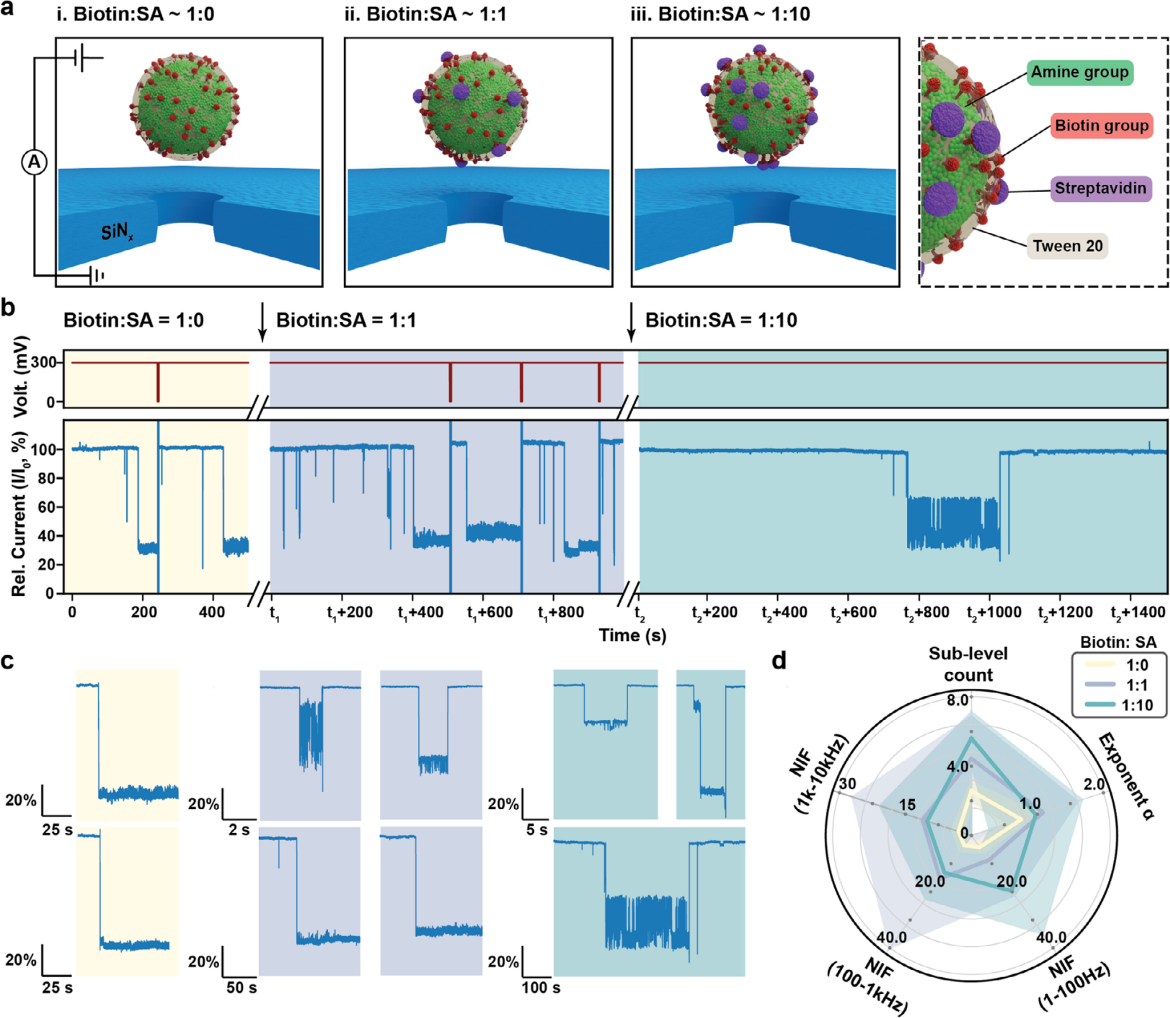
In situ monitoring of the surface chemistry transformation modulated via streptavidin (SA) binding. a) The schematic representation of measurement conditions including trapping of i) Amine‐Biotin‐Tween 20 functionalized silica nanoparticles (SiO_2_‐Biotin‐Tween nanoparticles) on SiN_x_ nanopore, ii) partially SA‐modified SiO_2_‐Biotin‐Tween nanoparticles after the addition of SA to the previous nanoparticles with concentration ratio close to 1:1, and iii) more densely SA‐modified nanoparticles with further addition of 10 times‐more concentrated SA. The external voltages in regime ii and iii are applied with the same polarity as in regime i, which are not plotted to avoid redundancy. Inset on the right side shows a zoom‐in schematic of the SA‐modified SiO_2_‐Biotin‐Tween nanoparticles surface with indications of each functional group component. b) Voltage profiles (top) and normalized ionic current traces (bottom) under three measurement conditions, starting from only SiO_2_‐Biotin‐Tween nanoparticles (yellow), to SA‐modified nanoparticles with biotin:SA ratios of 1:1 (blue) and 1:10 (green). c) Representative trapping events extracted from the corresponding trace in b). d) Combined fingerprint plot of all three conditions. To compute the radar plot metrics, the numbers of nanoparticle trapping events used for different biotin:SA ratios are *n*(1:0) = 15, *n*(1:1) = 8, and *n*(1:10) = 5, respectively. The solid lines represent the mean value, and colored bands denote one standard deviation around the mean.

Following the validation of nanoparticle fingerprinting, we further demonstrate in situ monitoring of surface chemical transformation, specifically via streptavidin binding to the biotin‐functionalized nanoparticles (**Figure** **5**). To enable the streptavidin binding, SiO_2_ nanoparticles are sequentially modified with APTES and NHS‐PEG_4_‐Biotin to provide a surface with biotin linkers, and also coated with Tween‐20 to reduce the nonspecific adsorption (Figure 5a).^[^ ^58^ ^]^ Biotinylated particles were first characterized by nanopore trapping in the same experimental condition as above. Then, streptavidin solution was added stepwise to the cis chamber with two different stoichiometric ratios (biotin: streptavidin≈1:1 and 1:10), and trap‐release cycles were continued to record the evolving trapping behaviors.

Before streptavidin addition, particles showed long, constant blockade events and manual reversal of voltage was required to release the particle and recover the open‐pore state (Figure 5b, yellow). After the addition of streptavidin at approximately a 1:1 stoichiometric ratio, the trapping behavior became intermittent and less stable, although occasional long blockades remained (Figure 5b, blue). Increasing the streptavidin concentration by ≈10‐fold produced further change, as the trapping events became rare and highly flickering, and no sustained blockage event was observed over extended recordings (over 25 min) under constant voltage (Figure 5b, green). With a significantly excess amount of streptavidin addition (over 1:100), we observed virtually no stable trapping event over hour‐long measurements (Figure S11, Supporting Information). Representative events illustrating the progressive change of trapping behaviors are shown in Figure 5c.

These transitions on trapping behavior indicate reduced capture efficiency and change in the character of ionic conductance perturbations during nanoparticle‐nanopore interaction. With the specific biotin‐streptavidin binding, association of streptavidin on the exposed biotin termini alters the nanoparticle surface by sterically shielding and neutralizing the amine‐associated positive charge,^[^ ^59^, ^60^ ^]^ and even reversing the net charge in certain scenarios such as the high excess concentration here, since streptavidin carries a net negative charge under the experimental buffer conditions.^[^ ^61^ ^]^ Such effects could diminish the EP force that initially enabled particle capture and trapping, thus reducing the trapping efficiency. In the meantime, streptavidin binding also increases local heterogeneity and structural complexity on the particle surface, since the binding brings more transient steric hindrance and charge inhomogeneity.^[^ ^62^ ^]^ This structural change therefore produces transient, configuration‐dependent perturbations to the ionic flow, resulting in the less stable and flickering trapping events after streptavidin binding (Figure 5c).^[^ ^63^ ^]^ Previous nanopore studies on biotin‐streptavidin complex have likewise attributed such rapid, irregular current fluctuations to the multiple docking confirmation.^[^ ^62^ ^]^ Moreover, existence of metastable binding states in the biotin‐streptavidin complex was also reported with the high‐speed force spectroscopy,^[^ ^62^ ^]^ suggesting that transient binding intermediates also modulate the local ionic conduction.

We also quantified these effects using the multi‐metric fingerprint, as shown in Figure 5d. As the ratio of streptavidin increases, average number of sub‐levels per event rises, which correlates with the emergence of more trapping configurations between particle and the pore. The low‐frequency exponent shows moderate increase after adding streptavidin, consistent with the increased structural complexity and slow reorganization process upon specific binding of protein.^[^ ^59^ ^]^ Band‐integrated NIFs also increase across three frequency ranges with the introduction of streptavidin, indicating the appearance of slow ionic modulation and enhanced perturbations, which matches the above‐discussed structural features upon protein binding to the particle surface.

As such binding events represent a form of biomolecular dynamics, we briefly discuss the broader question of the suitability for studying the biomolecular dynamics with different nanopore traps. This suitability of a trapping platform would depend on its temporal resolution, whether the trap perturbs the status of analyte, and the sensitivity of the trap to conformational change, chemical or binding events inside the trap or at the interface. A previous work has achieved sub‐milisecond temporal resolution and multi‐hour trapping stability via a DNA‐origami‐assisted nanocavity, presenting a powerful tool for resolving the rapid conformational transitions of single protein molecules, especially the nucleotide‐binding‐dependent conformational heterogeneity.^[^ ^22^ ^]^ While the strong EOF, steric restrictions and proximity to DNA structure lead to confinement and alter the local forces that could potentially influence the native status of analytes. In comparison, the SSN trap reported in this work uses direct electrokinetic capture without additional nanostructures, and offers high sampling bandwidth (200 kHz in acquisition, 50 kHz in analysis) detection of transitions related to the nanoparticle surface layer. The electrical field or hydrodynamic flow could also induce perturbations and shift the nanoparticle surface functional layer from its native condition, while the results from different tested voltages indicate such perturbations are less dominant under our experimental conditions and are more surface layer‐dependent (Figure S10, Supporting Information). The multi‐metric electrical fingerprints in streptavidin‐binding also showed sensitivity to interfacial binding. By revealing the progressive reconfiguration on the nanoparticle surface, our SSN trap presents a different and complementary regime for studying the biomolecular dynamics to other reported work.

Together, the multi‐metric analysis enables mapping each stage of the streptavidin‐binding on nanoparticles with a distinct electrical fingerprint. The nanopore metrology therefore exhibits the label‐free and in situ detection and quantification of surface chemistry transitions with single‐particle sensitivity.

## 3 Conclusion

We established a solid‐state nanopore‐based multi‐metric metrology for profiling the surface functionalization of individual nanoparticles with a nanopore trap. By combining a trap‐release measurement protocol with a multi‐metric analysis workflow that combines current blockage, sub‐level components, and frequency‐domain signal analysis, we distinguished silica nanoparticles modified with APTES, PEG_4_‐Biotin, and Tween‐20, respectively. Each species generates a unique electrical fingerprint through the particle trapping behaviors, which are linked to its surface charge, structural complexity, and intermolecular interactions. We further demonstrated in situ detection of specific streptavidin binding on biotinylated nanoparticle surfaces and resolved different stoichiometric ratios through the electrical fingerprints.

This methodology expands the scope of our label‐free, single‐particle nanopore sensing platform to surface chemistry characterization. With further refinement in electrical feature extraction and the use of low‐noise nanopore devices, more precise differentiation of nanoparticle surface coatings and live tracking of surface molecular interactions could be realized, offering powerful tools for applications in emerging contaminants, nanomedicine, targeted delivery, and point‐of‐care diagnostics.^[^ ^64^, ^65^, ^66^ ^]^

## 4 Experimental Section

### Nanoparticle Synthesis and Surface Modification

*SiO_2_ nanoparticles*: SiO_2_ nanoparticle was prepared with a well‐established alkaline catalysis sol‐gel method with minor modification.^[^ ^67^ ^]^ Ammonium chloride–ammonia (NH_4_Cl–NH_3_) buffer solution at pH 9.0 was prepared by adjusting 10 mM NH_4_Cl with aqueous ammonia. A total of 350 mL of the buffer was transferred into a 500 mL round‐bottom flask and heated to 60 °C with an oil bath. Upon reaching the target temperature, a mixture containing 100 mL of tetraethyl orthosilicate (TEOS) and 50 mL of cyclohexane was added to the flask. The resulting two‐phase system was vigorously stirred to form an emulsion and maintained under stirring for 24 h. After cooling to room temperature, the nanoparticle dispersed in aqueous phase was collected with separating funnel and collected by centrifugation with 16 000 rpm.

*SiO_2_─NH_2_ nanoparticles*: The particle solution was repeatedly redispersed in absolute ethanol and centrifugated three times and finally dispersed in 99% ethanol. (3‐Aminopropyl)triethoxysilane (APTES) was then added into solution with 1% concentration under vigorously stirring for 2 h. The solution was then worked up with repeating centrifugation and redispersed in 99% ethanol for three times and finally dispersed in water. The solution was further purified with dialysis in ultrapure water and the concentration was adjusted into 1% w/w.

*Biotinylated SiO_2_─NH_2_ Nanoparticles (SiO_2_‐Biotin Nanoparticles)*: EZ‐Link NHS‐PEG4‐Biotin kit (Thermo Fisher Scientific Inc.) was applied for biotinylation of SiO_2_─NH_2_ nanoparticles. In brief, 20 mM NHS‐PEG_4_‐Biotin was prepared as a stock solution. 1 mL 0.1% SiO_2_─NH_2_ nanoparticles were mixed with 10 µL of NHS‐PEG_4_‐Biotin solution in a vigorously stirred phosphate buffer (10 mM, pH∼8) and the reaction was left overnight. The solution was then purified with Amicon Ultra Centrifugal filters (10 kDa) to remove unreacted reagent and buffer and restore the particle concentration by addition of ultrapure water. The solution was further diluted 10 times before adding into the measuring buffer.

*Tween‐20‐Coated SiO_2_─NH_2_ Nanoparticles (SiO_2_‐Tween Nanoparticles)*: Tween‐20‐coated SiO_2_─NH_2_ nanoparticles were synthesized by adding Tween‐20 (10% v/v, 240 µL) to a solution of SiO_2_─NH_2_ nanoparticles (0.1% 60 mL). After vigorously stirring for 20 min, 3 mL of 1 M KCl solution was added into the solution and then stirred overnight. The solution was purified afterwards with dialysis to remove saturated Tween‐20.

After the synthesis of various surface‐functionalized nanoparticles, the Zeta potential values for each particles were measured with a zeta‐sizer (Nano ZS, Malvern Instruments) in the deionized water under pH∼6, the results were shown in Figure S12 (Supporting Information).

### Nanopore Fabrication

The silicon nitride (SiN_x_) nanopore devices used in this study were fabricated in wafer‐scale, starting from the standard 4‐inch Boron‐doped thick silicon wafers (Si, 380 ± 10 µm in thickness) supplied by the EPFL Center of MicroNanoTechnology (CMi), followed by deposition of a 60 nm‐thick dry silicon oxide (SiO_2_) layer and a 20 nm‐thick low‐stress silicon nitride (SiN_x_) layer on both sides of the wafer. Photolithography and dry etching were done in the CMi cleanroom facility to open apertures in the backside SiN_x_ layer to enable the subsequent wet etching process in Si/SiO_2_ layer, which led to the formation of a suspending SiN_x_ membrane (30 µm x 30 µm in size, 20 nm‐thick) on the front side. Acid piranha solution cleaning followed by baking under 300 °C was adopted to allow a clean and easy‐to‐wet nanopore surface, as described in the previous work.^[^ ^68^, ^69^ ^]^ Each chip was then cleaved manually out from the wafer, and the nanopore was produced in a well‐controlled way through transmission electron microscope (TEM) drilling on the SiN_x_ membrane. The pore sizes used in this study are in the 6–10 nm range in diameter, with a typical effective pore length of ≈8.7 nm.^[^ ^70^ ^]^

### Nanoparticle Trapping Experiment & Data Acquisition

The SiN_x_ nanopore chip was usually treated with oxygen plasma to activate the surface and facilitate the wetting. It is then assembled into a customized PMMA flowcell, and the details could be found in our previous work.^[^ ^71^ ^]^ The flowcell is composed of two parts in which the chambers (*cis* and *trans)* are connected only via the nanopore. Both chambers were filled with filtered 1 M KCl solution (pH∼6.5). The Ag/AgCl electrodes were inserted in each chamber, through which the voltage bias was applied and the ionic current was collected. The nanopore size was first checked by extracting the estimated pore size from the current‐voltage characteristic based on a conductance model and comparing with the TEM image.^[^ ^70^ ^]^ Blank ionic traces were also measured before adding nanoparticles to ensure the device was functioning normally and avoid measurement artifacts that could be caused due to contaminations on the nanopore chip or in the flowcell. Practically, our SSN trap consists of standard chips with SiN_x_ membranes and TEM‐drilled pores, whereas previously reported trap requires liquid bilayer coating on the drilled SiN_x_ nanopore and subsequent DNA‐origami folding and docking onto the passivated pore.^[^ ^22^ ^]^ This additional preparation step trades fabrication complexity for a sealed hydrodynamic nanocavity, enabling label‐free analysis of single protein molecules irrespective of their charges. The nanoparticles were then introduced in the *trans*‐chamber, where an active positive voltage was applied to initiate the gating. In the streptavidin experiment, the protein was well mixed with nanoparticles in the chamber. While changing to different measurement conditions, at t1 and t2 timepoints, streptavidin diluted with 1 M KCl were directly pipetted to the vicinity of the nanopore membrane and mixed gently with the nanoparticles in solution. The concentration ratio was estimated accordingly. The data was originally acquired using Axopatch 200B (Axon Instrument, USA) low‐pass filtered at 100 kHz with a built‐in Bessel low‐pass filter and sampled at a 200 kHz sampling rate, and then low‐pass filtered at a cutoff frequency of 50 kHz for subsequent analysis.

### Multi‐Metric Data Processing Workflow

After obtaining the continuously recorded raw data file, each independent gating event was first extracted according to the voltage protocol of each trap‐release cycle. A current threshold which is tied to the open‐pore baseline was then applied in each event to exclude false positives and to assist the segmentation. To do this, the open‐pore baseline was first verified by comparing with the reference current baseline before adding any nanoparticle samples. The whole event was then normalized by the mean value of open‐pore current. From the relative current, the mean value *I*_0_ and root‐mean‐square (RMS) value σ_*open*_ were calculated through the open‐pore segment. An event is considered gated when the current drops below *I*_0_ − Δ*I_th_*, where we chose Δ*I_th_* = 3σ_*open*_. After segmenting the open and gated‐pore parts from the event, histograms were plotted and fitted with GMM: for open‐pore segment, the number of components should be 1, otherwise the event needs to be removed due to potential baseline drift or false positive events that could not exceed the threshold; moreover, the mean value of this component was compared with 100% and a correction factor was applied to adjust the normalized current. For the gated‐pore region, the number of components was stored as the sub‐level state count to reflect the distribution of gated‐pore states as well as the particle‐pore interactions. Bayesian Information Criterion (BIC) and R‐squared values were adopted to ensure optimized automatic fitting and avoid overfitting (example results in Figure S4, Supporting Information). Noise characteristics were performed based on the PSD using Welch's method.^[^ ^43^ ^]^ The low‐frequency exponent α was fitted as detailed in Section 2.2 for gated‐pore to reflect possible slow processes related with surface kinetics. NIFs over three different frequency domains were calculated by comparing the integrated band noise of gated‐pore with open‐pore segment, to highlight the ionic‐flow barriers and dielectric fluctuations introduced by various coating and therefore add more dimensions in the electrical signature for better differentiation. The metrics from all events were finally summarized with mean and standard deviation value as statistical representation and compared across nanoparticle types in a radar‐plot. The data processing workflow was done using the Python‐based toolkit, and code is available on request.

## Conflict of Interest

The authors declare no conflict of interest.

## Author Contributions

N.C., T.‐H. C., and A.R. conceived the idea and designed the study. Y.T. fabricated the SiN_x_ nanopore substrates. N.C. fabricated the nanopore devices. T.‐H.C. performed the synthesis of nanoparticle samples and TEM imaging analysis. N.C. and Y.T. performed TEM characterizations of the nanopore device and nanoparticle samples. N.C. and T.‐H.C. performed the experiments. N.C., T.‐H.C., and A.R. wrote the first draft of the manuscript. A.R. supervised the work. All authors contributed to the writing and discussion of the manuscript.

## Supporting information



Supporting Information

## Data Availability

The data that support the findings of this study are available from the corresponding author upon reasonable request.
