# Supplementary material for: Nanopore Trap for Label‐Free Fingerprinting of Surface‐modified Single Nanoparticles
Source: Small Methods. 2025 Dec 9;10(3):e01765. doi: 10.1002/smtd.202501765 (PMC12893298; doi:10.1002/smtd.202501765)
Supplement: Supplementary file 1 — Supporting Information [file SMTD-10-e01765-s001.pdf]

Supporting Information for

Nanopore trap for label-free fingerprinting of surface-modified single nanoparticles

Nianduo Cai[1], Tzu-Heng Chen[1,*], Yunfei Teng[1,2], Akhil Sai Naidu[1] & Aleksandra Radenovic[1,2,*]

1. Laboratory of Nanoscale Biology, Institute of Bioengineering, Ecole Polytechnique Federale de Lausanne (EPFL), CH-1015 Lausanne, Switzerland
2. NCCR Bio-inspired Materials, Ecole Polytechnique Federale de Lausanne (EPFL), CH-1015 Lausanne, Switzerland

∗ To whom correspondence should be addressed;
E-mail: tzu-heng.chen@epfl.ch, aleksandra.radenovic@epfl.ch

Table of Contents

Figure S1. Current-voltage characteristics of the devices used in this study.

Table S1. Summary of the nanopore devices used and corresponding nanoparticle trapping experiments carried out on each nanopore device.

Figure S2. TEM images and size distribution of SiO_2 and SiO_2-NH_2 nanoparticles.

Figure S3. Complete ionic current recording of the stable long-gating event.

Figure S4. Summarized workflow of the nanopore metrology and examples of analysis results during the data processing for each type of nanoparticles.

Table S2. Details of the multi-metric statistical results as nanoparticle fingerprints.

Figure S5. Translocation behaviors of DNA molecules under open- and gated-nanopore states.

Figure S6. Characteristic events, histogram and fingerprint plot of Tween-20-coated SiO_2-Biotin nanoparticles.

Figure S7. Trapping behaviors or Tween-20-coated SiO_2-Biotin nanoparticles using two pores with different sizes.

Figure S8. Voltage-modulated gating behaviors showing the voltage-driven trap-release of nanoparticles and its dependence on the surface layer.

Figure S9. Noise spectrums from gating levels under different voltages for SiO_2-Biotin and SiO_2-Biotin-Tween nanoparticles.

Figure S10. Electrical fingerprints under different voltages for SiO_2-Biotin and SiO_2-Biotin-Tween nanoparticles.

Figure S11. Complete recording of ionic current profile with overdosed streptavidin addition in SiO_2-Biotin nanoparticles.

Figure S12. Zeta potential of corresponding nanoparticles used in the study.

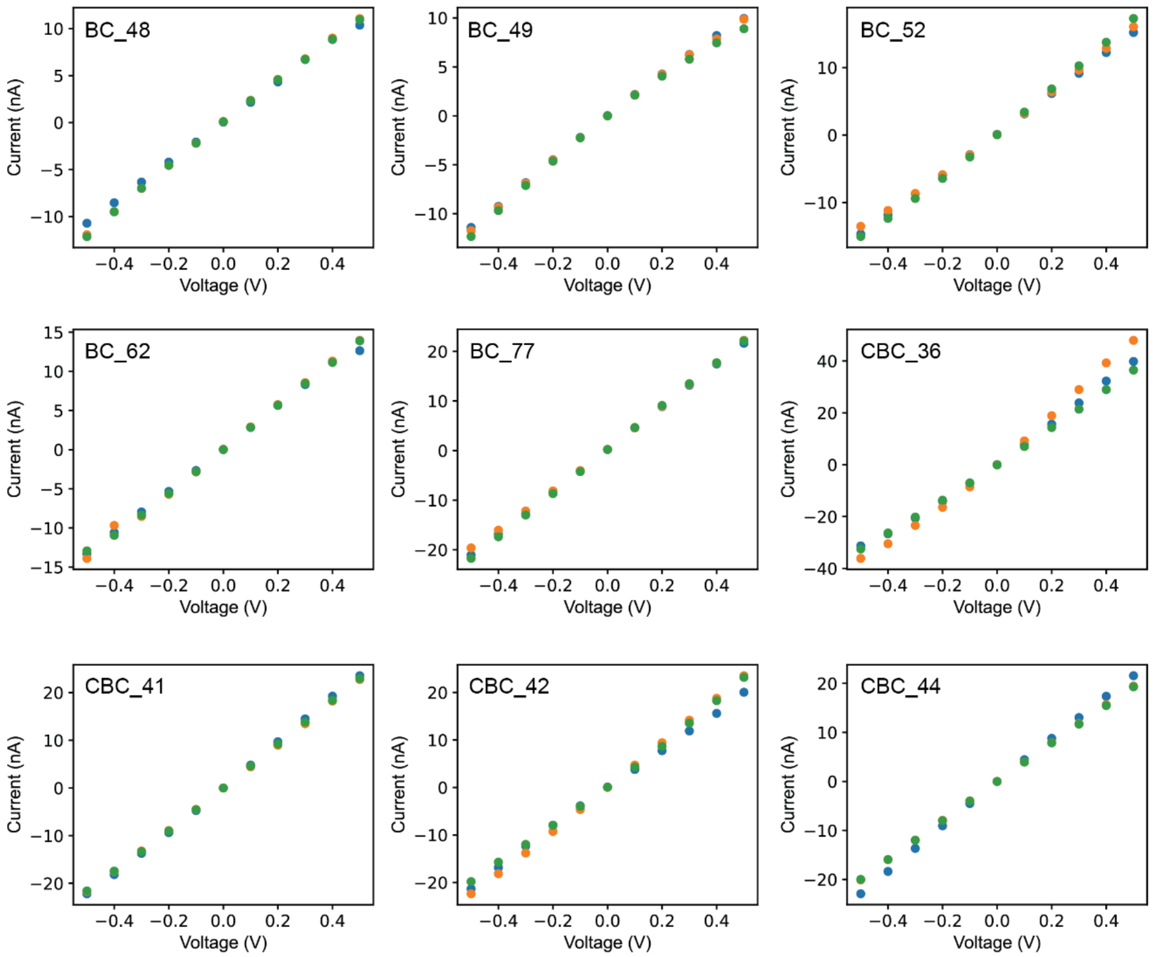

Figure S1. Current-voltage characteristics of all nanopore devices (N = 9) measured in 1M KCl solution by sweeping the voltage in the range of -500 mV to +500 mV. For each device, three individual IV characteristic tests are summarized in the plot with different colors (yellow, green and blue). The conductance values are extracted by a linear fit of each individual ohmic-like I-V responses, and nanopore diameters are extracted based on the conductance. Detailed values are presented in Table S1.

Chip ID	Nanoparticles measured	Conductance (nS)	Estimated nanopore diameter (nm)
BC_48	SiO_2-NH_2	22.5 ± 0.9	5.7 ± 0.1
BC_49	SiO_2-NH_2<br>SiO_2-Biotin	21.5 ± 0.1	5.9 ± 0.1
BC_52	SiO_2-Biotin<br>SiO_2-Biotin-Tween	30.8 ± 1.5	7.4 ± 0.2
BC_62	SiO_2-Tween	27.2 ± 0.5	6.8 ± 0.1
BC_77	SiO_2-Biotin-Tween,<br>+ Streptavidin	43.0 ± 0.9	8.8 ± 0.1
CBC_36	SiO_2-Biotin-Tween	75.9 ± 8.8	13.0 ± 1.0
CBC_41	SiO_2-Biotin-Tween,<br>+ Streptavidin	45.3 ± 1.0	9.2 ± 0.1
CBC_42	SiO_2-Biotin-Tween,<br>+ Streptavidin	43.2 ± 2.8	8.9 ± 0.4
CBC_44	SiO_2-Biotin-Tween	41.2 ± 3.0	8.7 ± 0.4

Table S1. Summary of the characteristics of nanopore devices used in this study and the nanoparticle trapping experiments that are carried out corresponding to each device. The open-pore conductance of each nanopore is measured using 1M KCl (pH~6.5) solution and extracted from the current-voltage characteristics (Figure S1). Based on the measured conductance, nanopore sizes are estimated according to the conductance model.[1] For each device, three individual IV tests are summarized to provide the mean and standard deviation values of the measured conductance and nanopore diameters. The estimated nanopore diameters are compared with TEM

images obtained for each nanopore device to confirm the working condition, before the addition of nanoparticle samples.

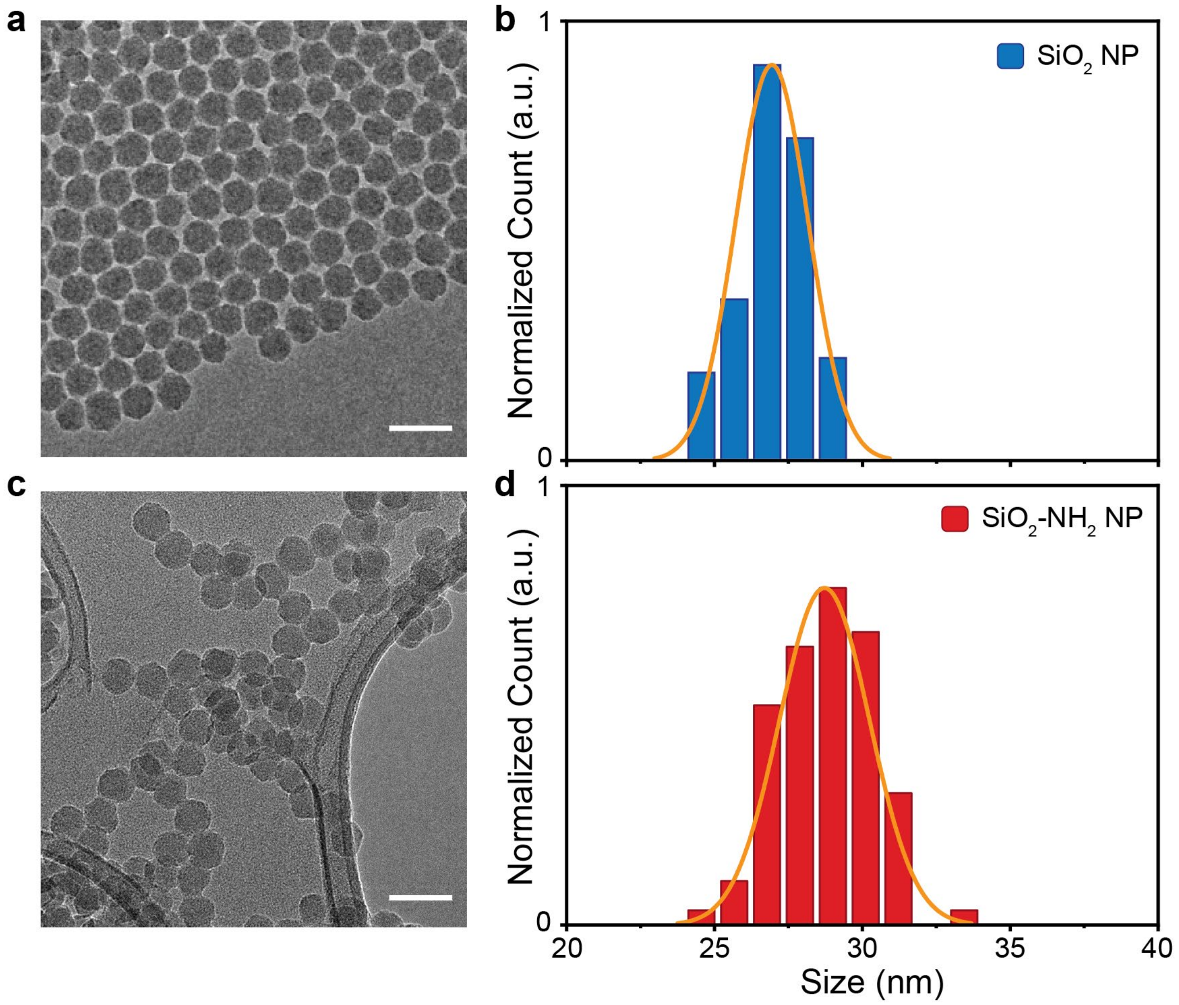

Figure S2. Transmission electron microscopy (TEM) image of (a) SiO_2 and (c) SiO_2-NH_2 nanoparticles, and corresponding statistics of size distribution (b and d) based on measurements of over 100 particles for each type. The SiO_2 nanoparticles serve as the common core for different types of surface modification in this work, which have a general size distribution between 25-30 nm in diameter. After the modification with APTES, the SiO_2 nanoparticles with compact -NH_2 surface groups exhibit an average particle size increase of approximately 2 nm in diameter. Scale bars are 50 nm.

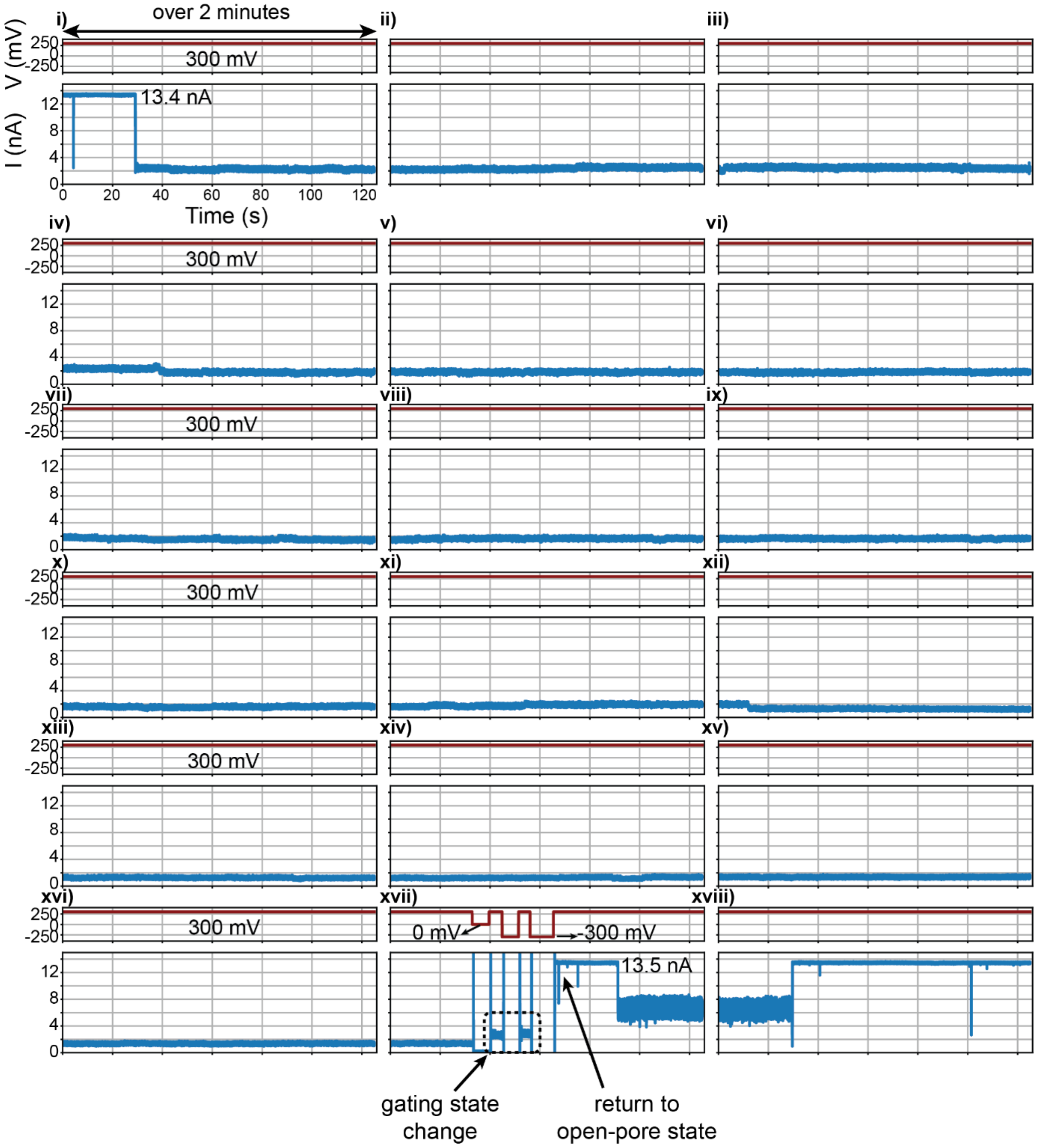

Figure S3. Complete voltage and ionic current recordings of the long-gating event in main text Figure 2b. Each subplot lasts for above 2 minutes. (i) The voltage is initially applied at 300 mV to enable a trapping event of Tween-20-coated SiO_2-Biotin nanoparticles (device BC_77), which starts after around 25 seconds. (ii-xvi) The gating event is maintained at a constant voltage of 300 mV for over 30 minutes, with a stable blockage level. (xvii-xviii) The nanoparticle is finally released after manually reversing the voltage, as the ionic current is restored to open-pore level like before the trapping. The voltage is initially reset to 0 mV to check if the nanoparticle can be released, even though the nanoparticle gating state has changed but it is not completely removed, as indicated by the ionic current after re-applying 300 mV. Similar behavior happens after

applying a first -300 mV reversed bias for a few seconds. The voltage-dependent trap-release process is further explored in Figure S8.

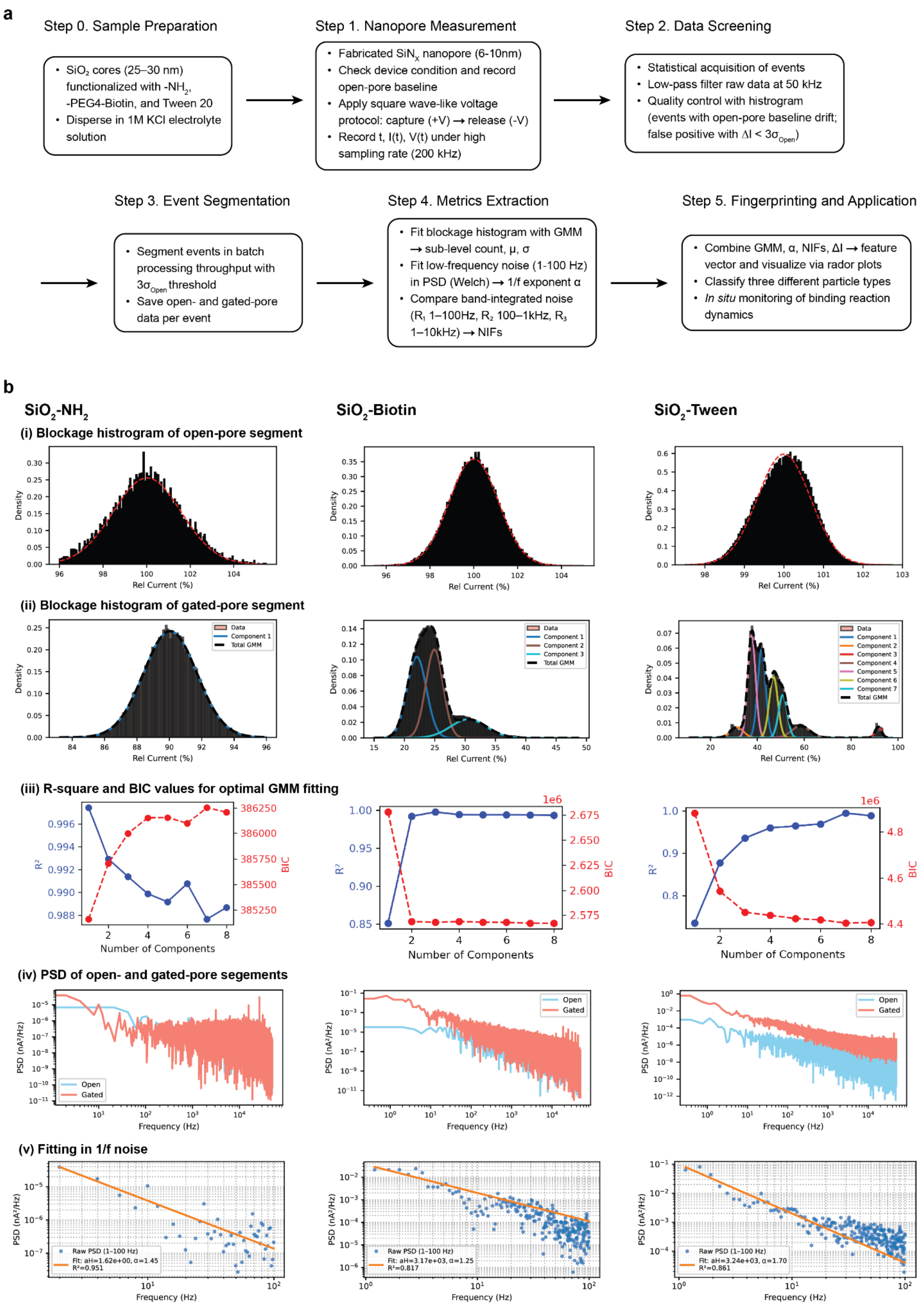

Figure S4. (a) Summary of the nanopore metrology workflow including data collection, QC screening, and automated post processing to generate electrical fingerprints for applications in differentiating nanoparticles and monitoring surface reaction dynamics. (b) Example outputs of the multi-metric analysis for each type of nanoparticles. As

introduced in the Experimental Section, a minor correction factor is applied to all relative currents to normalize the mean value from Gaussian fit to 1 (the red dashed-line in open-pore histograms). While leveraging Gaussian mixture model (GMM) in fitting the gated-pore histograms to obtain counts of sub-level states, Bayesian Information Criterion (BIC)[2] is usually applied to select the optimal number of components, which usually correspond to minimized BIC value. The R-squared values are referenced to avoid over-fitting that may happen in BIC with unlimited component numbers. Fitting in the low-frequency noise regime is done using Equation (2) to extract the exponent α, which also bases on R-squared value to evaluate the fitting goodness.

Metrics	Type of nanoparticle	Mean	Std
**Sub-level count**	SiO_2-NH_2	1.00	0
**NIF (1-100 Hz)**		0.92	0.55
**NIF (100-1 kHz)**		0.94	0.19
**NIF (1-10 kHz)**		1.03	0.15
α **exponent**		0.80	1.04
**Sub-level count**	SiO_2-Biotin	3.13	1.81
**NIF (1-100 Hz)**		14.68	19.48
**NIF (100-1 kHz)**		2.68	1.04
**NIF (1-10 kHz)**		1.67	0.46
α **exponent**		1.37	0.73
**Sub-level count**	SiO_2-Tween	4.10	2.17
**NIF (1-100 Hz)**		9.20	15.00
**NIF (100-1 kHz)**		3.38	2.27
**NIF (1-10 kHz)**		2.23	1.36
α **exponent**		1.93	1.05

Table S2. Detailed results of the multi-metrics from statistical analysis for each type of nanoparticle, which are obtained from 20-40 gating events and used for fingerprinting the three types of nanoparticles.

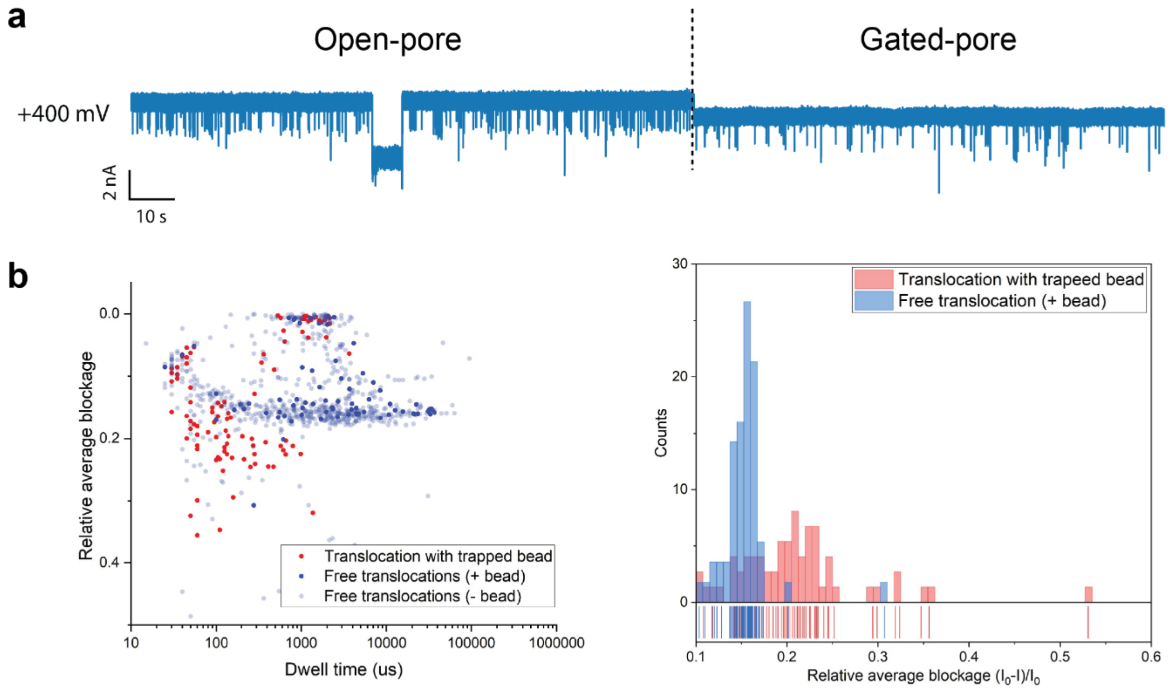

Figure S5. Translocation behaviors of 20 kbp DNA molecules at 400 mV under open/gated nanopore states, using nanopore device BC_48 and SiO_2-NH_2 nanoparticles. Representative ionic current traces are shown in (a). After addition of nanoparticles into the chamber but before trapping, translocations of DNA were observed. Upon nanoparticle trapping on the pore, translocation events were continuously observed under the gated-pore condition. The relative current blockage and dwell time distributions were analyzed for translocation events occurring in different regimes, including free translocation under open-pore state before (-bead) or after (+bead) adding nanoparticle sample, and translocation with trapped nanoparticle (represented as 'trapped bead'). In general, translocation events show higher average relative blockage and become faster under the gated-pore state. This probably suggests that with nanoparticle trapping on the pore, the effective pore area becomes smaller, and in the meantime the electrical field around the pore is enhanced, which together result in higher relative blockage and accelerated DNA translocation.[3,4]

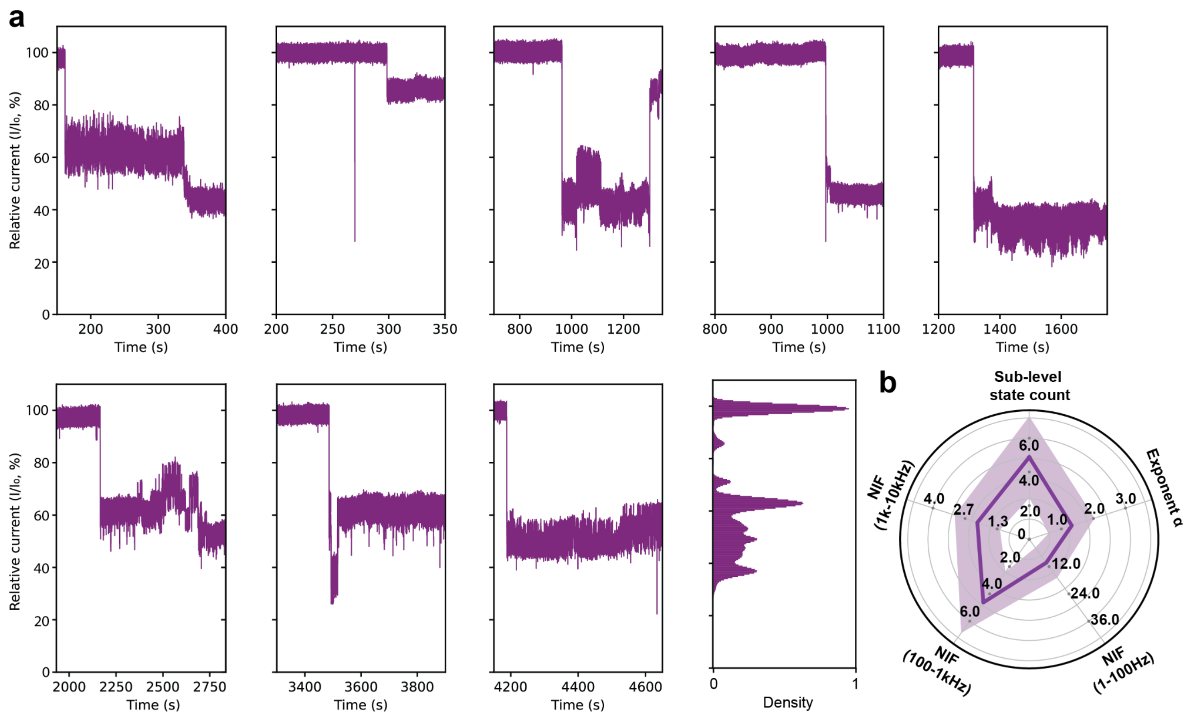

Figure S6. (a) 8 characteristic gating events of Tween-20-coated SiO_2-Biotin (SiO_2-Biotin-Tween) nanoparticles and summarized histogram of relative currents. (b) The radar-plot electrical fingerprint obtained following the analysis workflow, in the same scale as in the main text Figure 4. The SiO_2-Biotin-Tween nanoparticles exhibit combined gating behaviors of SiO_2-Biotin and SiO_2-Tween nanoparticles, including a wide range of blockage levels, occasional hopping between multiple levels and generally more dynamic and flickering gating levels. Similar trends of gating dynamic changes are exhibited before and after coating Tween-20, either on the SiO_2-Biotin nanoparticles (i.e., SiO_2-Biotin vs. SiO_2-Biotin-Tween nanoparticles), or on the SiO_2-NH_2 nanoparticles (i.e., SiO_2-NH_2 vs. SiO_2-Tween nanoparticles), consistent with the surface dynamics introduced via non-covalently coated Tween-20 layer on the particle surfaces.

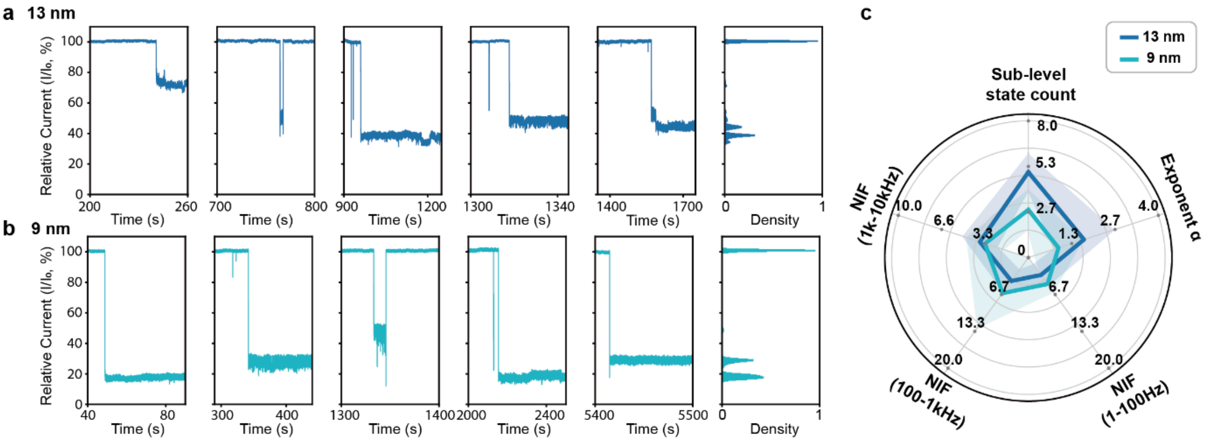

Figure S7. Trapping behaviors of Tween-20-coated SiO_2-Biotin nanoparticles using two nanopores with different sizes, including 5 characteristic gating event traces from pores of (a) d_{pore} ~ 13 nm, (b) d_{pore} ~ 9 nm, and (c) the corresponding radar-plot fingerprints (n = 5). By comparing the trapping of same Tween-20-coated SiO_2-Biotin particles on two pores with distinct sizes and thus nanopore-to-nanoparticle size ratio, the smaller pore (~9 nm) produces deeper blockages, less sub-level states and higher low-frequency exponent compared with larger pore (~13 nm).

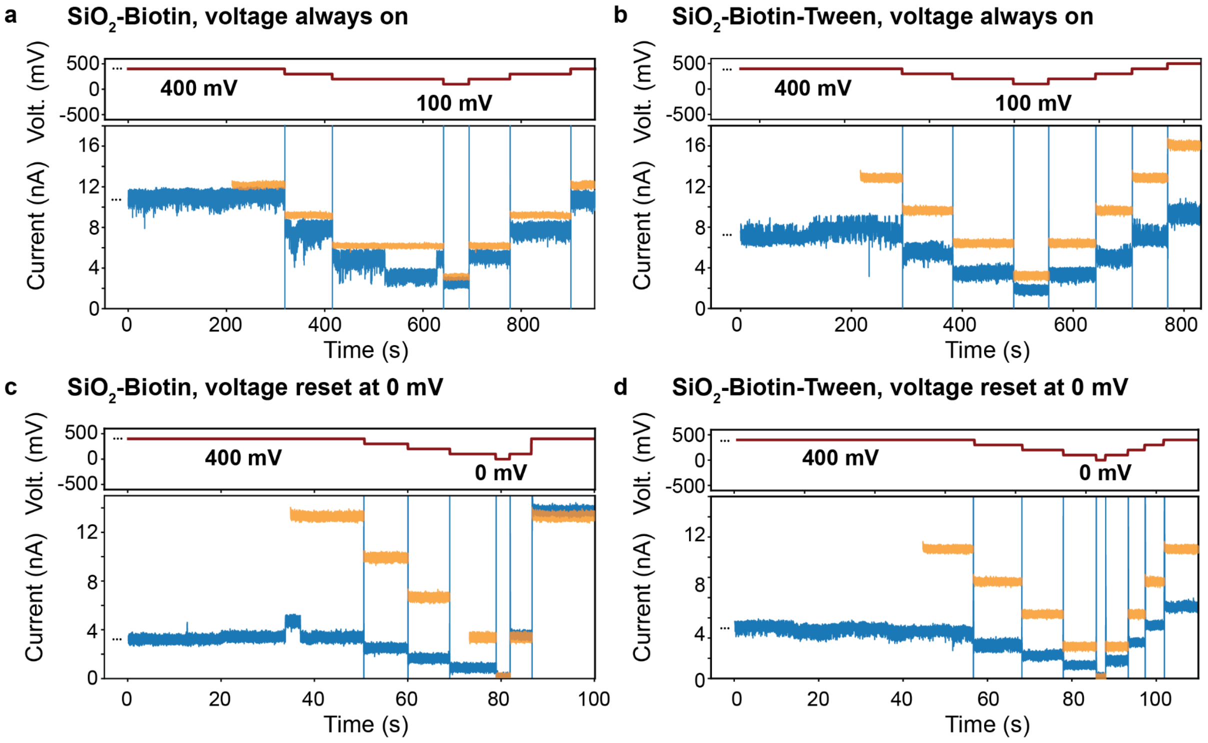

Figure S8. Voltage-modulated gating behaviors with two different voltage protocols (dark red): (a, b) gradually reducing the voltage from 400 mV to 100 mV and increasing back to 400 mV again, and similarly (c, d) reducing the voltage from 400 mV to 0 mV and then increasing to 400 mV, instead of halting at 100 mV. Two types of surface-modified nanoparticles are tested under these voltage protocols: SiO_2-Biotin nanoparticles (a, c), and Tween-20-coated SiO_2-Biotin nanoparticles (b, d). The exhibited ionic current traces (blue) start from the middle of the trapping events, with the initiation of each event and the stability check (which usually lasts from 30 seconds to a few hundred seconds) omitted for visual simplicity and clarity. For comparison and better visualizing the nanopore state, the open-pore baseline currents corresponding to each voltage level are overlaid on the plot (orange). For SiO_2-Biotin nanoparticles, the gating state remained almost the same while modulating the voltage while always keeping the voltage on. However, the nanoparticle can be quickly released after a short voltage reset at 0 mV, and the pore is restored to its open-pore state. While for Tween-20-coated SiO_2-Biotin nanoparticles (SiO_2-Biotin-Tween nanoparticles), it remains trapped under both voltage protocols. Similar to the SiO_2-Biotin nanoparticle, the Tween-20-coated nanoparticle remains in a relatively stable gating state as the voltage changes, provided it is not reset to 0 mV, as indicated by the blockage current and relative current level compared to the open-pore baseline. Although the nanoparticle is not released after reset voltage, the gating state changed after re-applying the voltage based on the relative current, which suggests a change in local geometry or conformation after voltage reset. Possible cause of no-release for the Tween-20-coated nanoparticles could be related with the slow adsorption/desorption dynamics of Tween-20 or the complex interactions between the long chains in the surface layer and the nanopore.[5,6] The result has demonstrated that gating behaviors

are voltage-dependent, as they suggest that the voltage-induced electrophoretic (EP) force and electro-osmosis flow (EOF) are the driven forces to facilitate and modulate the trapping state, which further validates the gate-dock-release voltage cycling protocol. In the meantime, it also shows that the surface layer determines the pore-particle interaction, and this in turn proves the sensitivity of the nanopore metrology in differentiating nanoparticles.

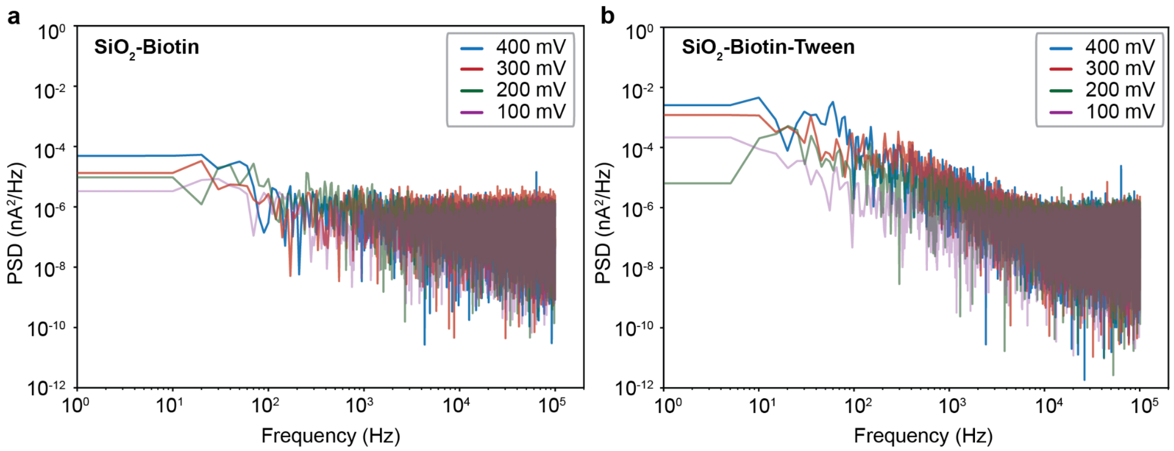

Figure S9. Noise analysis of gating levels under different voltages. Power spectrum density (PSD) are plotted for (a) SiO_2-Biotin, and (b) SiO_2-Biotin-Tween nanoparticles using the gated ionic current trace from Figure S8 (a) and (b) All PSD plots are overlapped for comparison and plot transparency gradually increases from 400 mV to 100 mV for better visualization. The results show generally similar noise spectrums under different voltages for each type of nanoparticles, with minor differences in low-frequency range where the noise power is positively proportional to the ionic current values and thus the voltages. On the contrary, major differences exist between the two types of nanoparticles in each frequency band. The noise analysis supports that the analysis workflow developed for nanopore metrology in this study is less dependent on experimental conditions such as voltages, while being more sensitive to nanoparticle surface chemistry.

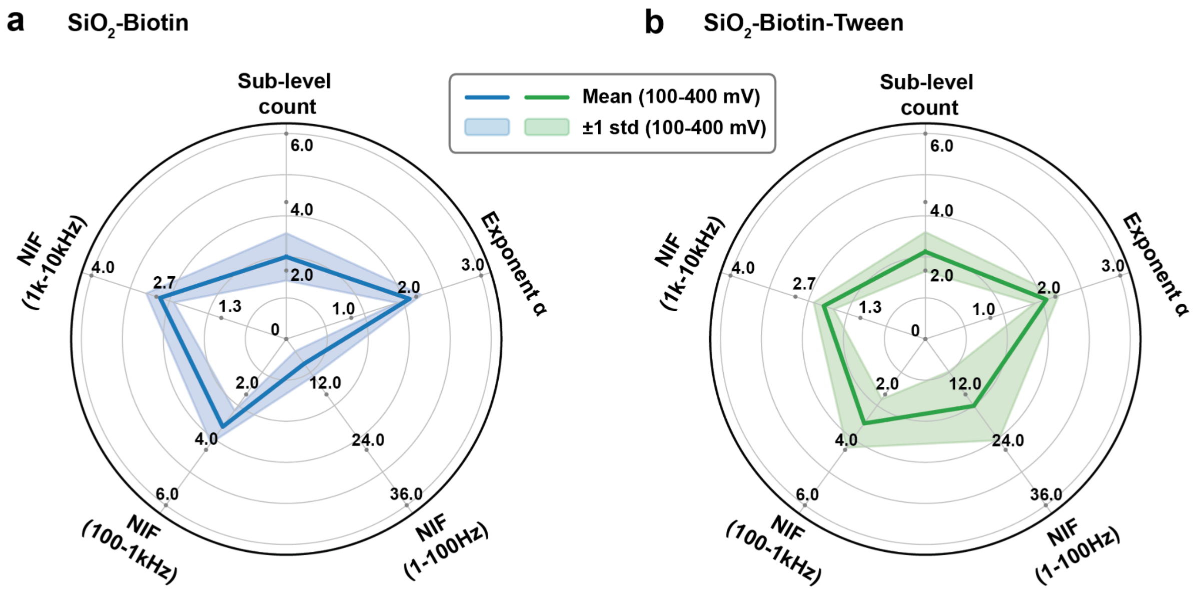

Figure S10. Electrical fingerprint under different voltages for (a) SiO_2-Biotin and (b) SiO_2-Biotin-Tween nanoparticles. Open-pore and gated-pore current signals were collected under 100-400 mV voltages as in Figure S8, which are used to generate the fingerprints for each voltage condition. The solid line represents each feature's mean value across 100-400 mV, and the colored band represents its one standard deviation around the mean. The narrow colored bands in both radar plots indicate small variations of each feature across different voltage conditions, which further supports that the fingerprint is less dependent on the experimental conditions.

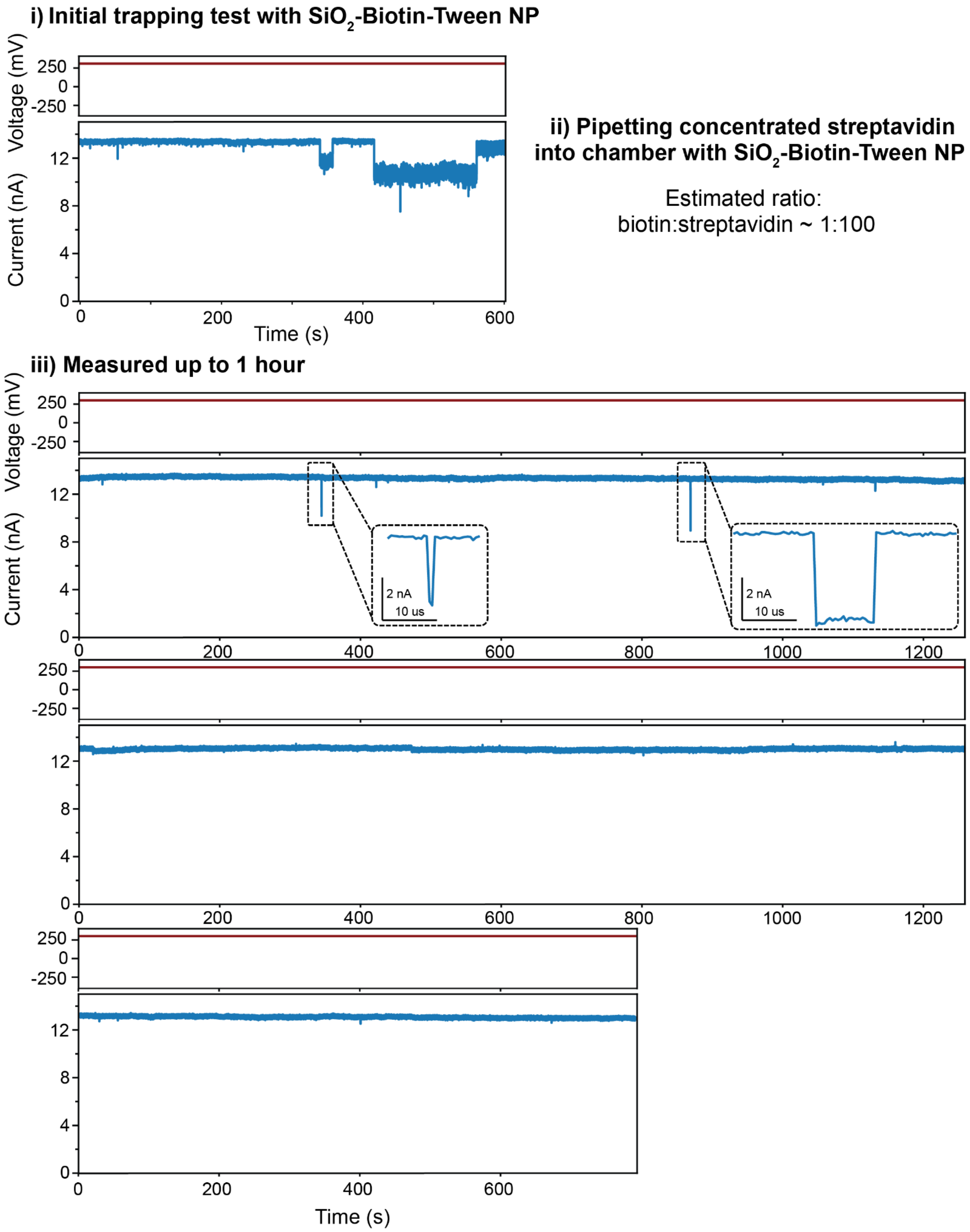

Figure S11. Recording of voltage and ionic current profiles from experiment (chip BC_77) with overdosed streptavidin addition into the biotin-terminated SiO_2-Biotin nanoparticles. Stable gating events have been observed initially with Tween-20-coated SiO_2-Biotin nanoparticles. The concentrated streptavidin is first diluted in an Eppendorf tube to a pre-concentration, which is then pipetted directly into the flow cell chamber loaded with nanoparticles. The final estimated biotin:streptavidin ratio is around 1:100, providing a streptavidin-overdosed condition. Recordings start

immediately after mixing, and within 1 hour of a sufficiently long recording, no stable trapping events occur, suggesting that streptavidin-biotin binding has indeed tuned the surface chemistry and disabled nanoparticle trapping at a high concentration of streptavidin.

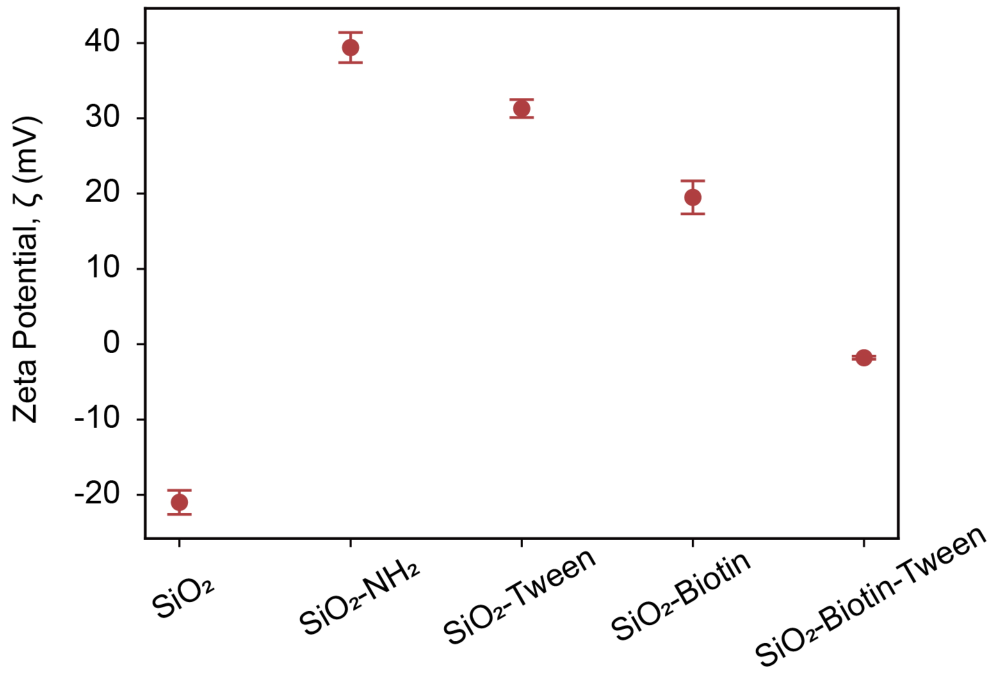

Figure S12. Zeta potential of the corresponding nanoparticles measured in deionized water with a pH value of around 6.0. Error bars are generated from three individual measurements for each type of nanoparticle.

References

1. Kowalczyk SW, Grosberg AY, Rabin Y, Dekker C. Modeling the conductance and DNA blockade of solid-state nanopores. Nanotechnology 2011;22(31):315101.

2. Bayesian Approach to Mixture Analysis [Internet]. In: Finite Mixture Models. John Wiley & Sons, Ltd; 2000 [cited 2025 Aug 26]. page 117–34.Available from: https://onlinelibrary.wiley.com/doi/abs/10.1002/0471721182.ch4

3. Wanunu M, Sutin J, McNally B, Chow A, Meller A. DNA Translocation Governed by Interactions with Solid-State Nanopores. Biophysical Journal 2008;95(10):4716–25.

4. Plesa C, van Loo N, Ketterer P, Dietz H, Dekker C. Velocity of DNA during Translocation through a Solid-State Nanopore. Nano Lett 2015;15(1):732–7.

5. Kovalchuk NM, Simmons MJH. Effect of Surfactant Dynamics on Flow Patterns Inside Drops Moving in Rectangular Microfluidic Channels. Colloids and Interfaces 2021;5(3):40.

6. Zhang Z, Orski S, Woys AM, Yuan G, Zarraga IE, Wagner NJ, et al. Adsorption of polysorbate 20 and proteins on hydrophobic polystyrene surfaces studied by neutron reflectometry. Colloids and Surfaces B: Biointerfaces 2018;168:94–102.
